# The Solubility Studies and the Complexation Mechanism Investigations of Biologically Active Spiro[cyclopropane-1,3′-oxindoles] with β-Cyclodextrins

**DOI:** 10.3390/pharmaceutics15010228

**Published:** 2023-01-09

**Authors:** Anna A. Kravtsova, Anna A. Skuredina, Alexander S. Malyshev, Irina M. Le-Deygen, Elena V. Kudryashova, Ekaterina M. Budynina

**Affiliations:** 1 Department of Chemistry, Lomonosov Moscow State University, 119991 Moscow, Russia; 2 Faculty of Medicine, Lomonosov Moscow State University, 119991 Moscow, Russia; 3 Dukhov Research Institute of Automatics (VNIIA), 127030 Moscow, Russia

**Keywords:** β-cyclodextrins, cyclopropanes, spirooxindoles, inclusion complexes, solubility, antibacterial activity

## Abstract

In this work, we first improved the aqueous solubility of biologically active spiro[cyclopropane-1,3′-oxindoles] (SCOs) via their complexation with different β-cyclodextrins (β-CDs) and proposed a possible mechanism of the complex formation. β-CDs significantly increased the water solubility of SCOs (up to fourfold). Moreover, the nature of the substituents in the β-CDs influenced the solubility of the guest molecule (MβCD > SBEβCD > HPβCD). Complexation preferably occurred via the inclusion of aromatic moieties of SCOs into the hydrophobic cavity of β-CDs by the numerous van der Waals contacts and formed stable supramolecular systems. The phase solubility technique and optical microscopy were used to determine the dissociation constants of the complexes (*K_c_*~10^2^ M^−1^) and reveal a significant decrease in the size of the formed crystals. FTIR-ATR microscopy, PXRD, and ^1^H-^1^H ROESY NMR measurements, as well as molecular modeling studies, were carried out to elucidate the host–guest interaction mechanism of the complexation. Additionally, in vitro experiments were carried out and revealed enhancements in the antibacterial activity of SCOs due to their complexation with β-CDs.

## 1. Introduction

**Figure 1:**
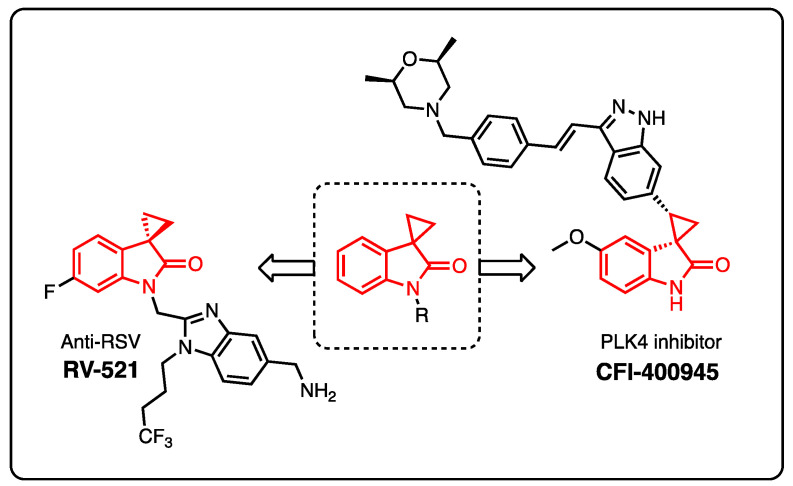
Examples of reported bioactive SCOs.

Spirocyclic compounds have important applications in medicinal chemistry due to the tetrahedral nature of the spiro-linked carbon. Namely, spirooxindoles serve as privileged scaffolds in drug discovery [1]. Many spirooxindole derivatives were found to play fundamental roles in biological processes and exhibit important pharmacological activities [2]. Meanwhile, the spiro-fusion of the oxindole scaffold with a cyclopropane unit allows for the creation of perspective drug candidates due to the enhancement of conformational rigidity, as well as chemical and metabolic stability [3]. For example, CFI-400945, a potent Polo-like kinase 4 (PLK4) inhibitor, and RV-521, a viral fusion inhibitor, advanced into Phase II clinical trials for the treatment of human cancers and RSV infection, respectively (Figure 1) [4,5]. Additionally, several compounds bearing spirocyclopropaneoxindole (SCO) scaffolds are currently under active development: namely, anti-HIV agents [6], bromodomain-containing protein 4 inhibitors [7], progesterone receptor antagonists [8], thyroid hormone receptor-beta agonists [9], AMP-activated protein kinase activators [10], serotonergic agents [11], and non-receptor tyrosine kinase inhibitors [12].

In drug development, aqueous solubility is a critical factor in substrate selection; up to 77% of screened compounds were reported to have inadequate solubility for subsequent testing. Poor aqueous solubility may be responsible for the decrease in the pharmacological effect and may cause other biological problems [13]. Supramolecular hosts, such as cyclodextrins (CDs), are widely used to improve the aqueous solubility and other properties of drug-like molecules, e.g., stability and bioavailability [14].

**Figure 2:**
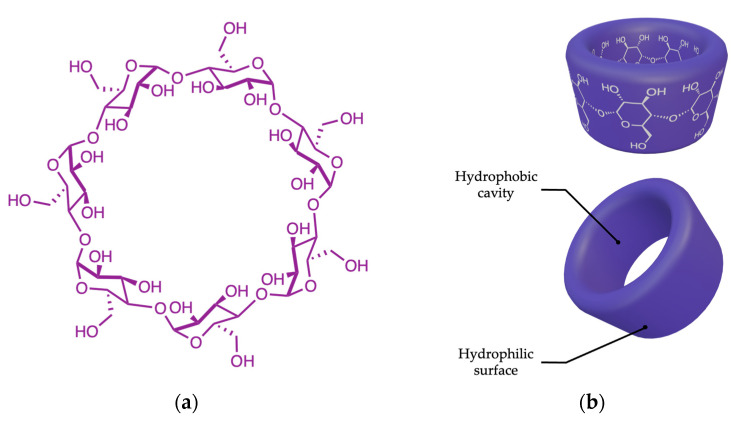
Schematic representations of (**a**) general chemical structure and (**b**) 3D structure of β-CD.

CDs are cyclic oligosaccharides containing five or more D-glucopyranose residues that are linked by α(1→4)-glycosidic bonds into a toroidal-shaped macrocycles (Figure 2). The external face of CDs is hydrophilic due to the presence of primary and secondary OH groups, whereas the internal cavity is relatively nonpolar [15]. These features of CD structures predispose them to encapsulate hydrophobic moieties, forming host-guest inclusion complexes and improving the guest molecule stability, solubility, and bioavailability [16].

The most common CDs, otherwise known as parent CDs, consist of six (α-CD), seven (β-CD), or eight (γ-CD) glucopyranose rings. The difference in the sizes of the inner cavity in the parent CDs steers them towards guest molecules with appropriate sizes and structures. For SCOs with aromatic fragments, β-CD is the most suitable host, as it has the most suitable cavity size for benzene ring entrapment [17,18]. Recently, CD derivatives with various substituents at the pyranose hydroxyl groups were used to increase the efficiency of complexation [19].

In this paper, we discuss an approach to improving the aqueous solubility of SCOs and, consequently, their biological activity via the formation of inclusive complexes with β-CDs. Therefore, 3′-Aryl-substituted SCOs **2** were synthesized as new model substrates to study the efficiency and the underlying mechanism of complexation with β-CDs.

## 2. Materials and Methods

### 2.1. Materials

The 2-hydroxypropyl β-cyclodextrin (HPβCD) and methyl β-cyclodextrin (MβCD) are both from Sigma-Aldrich (St. Louis, MO, USA). Sulfobutyl ether β-cyclodextrin sodium salt (SBEβCD) is from Zibo Qianhui Biotechnology Co. (Zibo, Shandong, China). Ethanol is from Reakhim (Moscow, Russia). Sodium phosphate buffer tablets for solution preparation were obtained from Pan-Eco (Russian Federation). *E. coli* ATCC 25922 is from the Russian collection of industrial microorganisms of the Kurchatov Institute, National Research Institute Centre.

### 2.2. Methods

#### 2.2.1. Synthesis of Investigated Compounds

##### General Information

NMR spectra were acquired at room temperature; the chemical shifts δ were measured in ppm with respect to solvent (1H: CDCl_3_, δ = 7.27 ppm; ^13^C: CDCl_3_, δ = 77.0 ppm). Splitting patterns are designated as s, singlet; d, doublet; t, triplet; m, multiplet; dd, double doublet. Coupling constants (*J*) are given in hertz. The structures of all compounds were elucidated with the aid of 1D NMR (1H, 13C) and 2D NMR (ROESY 1H−1H) spectroscopy. High-resolution mass spectra (HRMS) were performed using ESI and a TOF mass analyzer. Analytical thin-layer chromatography (TLC) was carried out with silica gel plates (silica gel 60, F254, supported on aluminum) and was visualized with a UV lamp (254 nm). Column chromatography was performed on silica gel 60 (230−400 mesh). NaH (60% dispersion in mineral oil) and trimethylsulfoxonium iodide are available commercially. Alkenes 1a–d and cyclopropanes 2a–d were prepared by Knoevenagel/Corey−Chaykovsky reactions [20,21], starting from the corresponding aldehydes, according to the published procedures [22]. Their spectra and physical data are consistent with earlier published data, except for the unreported compounds **1a,d** and **2a,d**. Their spectral and physical data are given in Supplementary Materials. All the reactions were carried out using freshly distilled and dry solvents.

##### General Procedure for the Synthesis of Alkenes 1

An aromatic aldehyde (1.1 equiv) was added to a solution of 1-methylindolin-2-one (1.0 equiv) in ethanol (1 M). The resulting solution was added dropwise to a solution of sodium hydroxide (2.0 equiv) in ethanol−water (1:2, 0.67 M) at 0 °C (ice bath). When the addition was completed, the reaction mixture was allowed to warm up to room temperature and was stirred for a specified time. Then, the reaction mixture was diluted with water and extracted with ethyl acetate. The organic layer was washed with water, dried with sodium sulfate, and concentrated under reduced pressure. Alkene 1 was purified by column chromatography on silica gel.

##### General Procedure for the Synthesis of Cyclopropanes 2

A suspension of sodium hydride (2.2 equiv) and trimethylsulfoxonium iodide (2.0 equiv) in DMF was stirred at room temperature for 30 min, then a solution of alkene 1 (1.0 equiv) in DMF was added dropwise. When addition was completed, the reaction mixture was stirred at room temperature for a specified time. Then, the reaction mixture was diluted with water and extracted with ethyl acetate. The organic layer was washed with water, dried with sodium sulfate, and concentrated under reduced pressure. The residue was washed with petroleum ether and dried.

### 2.3. Measurements

#### 2.3.1. Solubility Studies

The samples solubility was studied by shake-flask method, as performed in [23]. Briefly, 5 mg of the sample was added to 5 mL of buffer solution in a glass vial. The solutions were intensely stirred at 25 °C for 6 h to achieve thermodynamic equilibrium. For dissolution rate studies, the aliquots were taken in the time range from 0.5 to 6 h. As sedimentation time significantly influences equilibrium solubility, all samples were stored without stirring for 18 h. Then, the concentration of dissolved sample was analyzed by UV spectroscopy. Physical mixture (PM) was performed by mixing 2a and CD powders. In the case of the kneading method (KM), the mixture was grinded until a homogeneous mass formed.

The phase solubility studies were conducted according to the well-known Higuchi and Connons method [24,25,26]. The *K_c_* values of the complexes were calculated regarding the phase solubility diagram:

$$K_{c}=\frac{slope}{S_{0}\left(1-slope\right)}$$

where *S*_0_ is the solubility of **2**.

The experiments were carried out three times, and the values are presented with standard deviations.

#### 2.3.2. UV Spectroscopy

The UV spectra were recorded by a Ultrospec 2100 pro instrument (Amersham Biosciences, Germany), within a wavelength range from 200 nm to 450 nm in a 1 mL quartz cell Hellma Analytics (Müllheim, Germany). The concentration of 2 was determined using the intensity at 265 nm.

#### 2.3.3. FTIR Microscopy

The FTIR microscopy was performed by the Bruker LUMOS FTIR microscope (Bruker, Ettlingen, Germany). The FTIR spectra were recorded in 4000–800 cm^−1^ regions with 2 cm^−1^ spectral resolution in ATR mode. For each spectrum, 70-fold scanning and averaging were carried out. The background was recorded according to the measurement position. The spectra and images were analyzed by Opus 8.2.28 software. 

#### 2.3.4. Dynamic Light Scattering (DLS)

DLS was used to determine the size of the particles by a Zetasizer Nano S «Malvern» equipped with 4 mW He–Ne-laser 633 nm (Malvern Instruments, Malvern, UK). The experiments were performed three times for each sample at 25 °C, using the Correlator system K7032-09 Malvern (Worcestershire, UK) and the software Zetasizer Software (Malvern Instruments, Malvern, UK). The values are reported with standard deviations.

#### 2.3.5. Powder X-ray Diffraction Analysis (PXRD)

PXRD patterns of 2 and their complexes with CDs (7–10 mg) were recorded using a Rigaku SmartLab (Rigaku Corporation, Tokyo, Japan), equipped with Cu-X-ray anode tube in the scanning range 1.5–80.0° with a step size of 5°/sec. X-rays were generated with 60 kV and 1.5 kW.

#### 2.3.6. Minimum Inhibition Concentration (MIC)

The overnight culture was used for all in vitro experiments (the bacteria were grown in Luria Bertuni medium for 12 h). MIC was determined by agar well diffusion method [27]. Briefly, overnight bacteria (500 μL) were distributed over the solid agar surface (15 mL of Luria Bertuni medium) on Petri dishes. Four wells were incised in the medium by sterile plastic pipette tip (d = 10 mm). The 50 μL was put in the wells (three for the samples and one for negative control (sterile buffer)). The CD-SCO complexes were obtained by KM method (molar ratio 3: 1). In 20 min, the Petri dishes were placed into the incubator at 37 °C for 24 h. Then, the appeared inhibition zones were analyzed. The experiments were carried out three times. The MICs are reported with standard deviations.

#### 2.3.7. System Preparation

Methyl-β-CD (MβCD) and ligands structures were constructed using the 3D-sketcher module in Maestro (Schrödinger, 2021) and then submitted to 10,000 steps of Polak–Ribiere conjugate gradient energy minimization by means of the Macromodel software (Schrödinger, 2021), using the OPLS3e force field [28] and GB/SA model as solvation treatment [29]. Docking studies were performed with the Glide program [30], using the centroid of MβCD to centre the grid box as docking space. Docking poses with RMS deviation <0.5 Å were discarded, and at most five docking poses were retrieved and subjected to a visual analysis. The best scoring pose of each stereoisomer was selected for further modeling (Table S1; Supplementary Materials).

#### 2.3.8. Force-Field Parameterization

For MβCD and ligands, General Amber Force-Field 2 (GAFF2) parameterization [31,32] was chosen, and charges were assigned using the AM1-BCC method [33] with bond charge corrections. All parametrization procedures were made using the AnteChamber Python Parser Interface (ACPYPE) v. 2022.6.6 [34].

#### 2.3.9. System Preparation and Simulation of Molecular Dynamics (MD)

Each system, consisting of MβCD molecule (168 atoms) and docked ligand (approx. 50 atoms), was immersed in the cell, with 1 nm distance between the solute and the cell, and filled with TIP3P water model [35]. The system energy was optimized using the gradient descent algorithm (1000 steps). For solvent equilibration, both temperature and pressure coupling with 5000 steps were performed. Classical MD simulations of 50 ns (25 ∗ 106 steps with a step length of 2 fs) trajectories for each system were performed (Figure S2; Supplementary Materials). For all simulation routines, including energy minimization and equilibration, the GROMACS [36] (v. 2021.3) program package was used as MD engine.

## 3. Results and Discussion

### 3.1. Synthesis of SCOs ***2***

**Figure 3:**
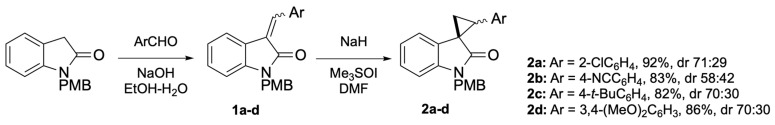
Synthesis of SCOs **2**.

Initially, we synthesized SCOs **2a–d** with various aryls at the three-membered ring via a two-step procedure starting from *N*-(*p*-methoxybenzyl)oxindole (*N*-PMB-oxindole) and the corresponding aromatic aldehydes (Figure 3). The synthetic sequence included Knoevenagel condensation and a Corey–Chaykovsky reaction [37,38]. This simple, cheap, and efficient method allows for a wide variation of substituents in the final SCOs by using various commercially available aldehydes and oxindoles. SCOs **2a–d** were obtained in good yields as diastereomeric mixtures.

### 3.2. Solubility of SCOs ***2***

As expected, SCOs **2a–d** are hydrophobic compounds due to the presence of three benzene rings in their molecules. Their intrinsic solubilities (*S*) were studied within two different aqueous media with the pH values of 7.4 (sodium phosphate buffer) and 2.0 (0.01 M HCl), simulating physiological conditions: blood plasma and stomach acid, respectively. In the UV spectra of the resulting solutions, wide absorption bands with maxima at ca. 265 nm were detected in all cases.

We found that the increase in *S* strongly correlates with the increase in the polarity of aryl substituents in SCOs **2**: *S***_2c_** < *S***_2a_** < *S***_2d_** < *S***_2b_** (Table 1). As the samples do not possess pH-sensitive groups, the changes in pH did not influence the *S* values in any noticeable way; thus, further experiments were conducted at neutral pH.

Herein, we obtained SCOs with four substituents, with different position patterns and electronic effects. Although these groups seemed quite similar (primary as hydrophobic ones), some of them could participate in other interesting intermolecular interactions: -Cl participates in halogen bonding and hydrophobic interactions[39], and -CN can form intermolecular H-bonds by N as acceptor and, less often, hydrophobic contacts [40].

Thus, cyclopropanes **2a** and **2b** were chosen as model compounds in this study.

### 3.3. Preparation of SCO 2–β-CD Complexes

β-CD complexes with SCOs **2** were prepared using several techniques [41,42]. Among them, the physical mixture approach (PM) and the kneading method (KM) were found to be the most efficient and reliable for highly hydrophobic compounds. Additionally, these approaches can affect the size and morphology of the formed particles [41].

First, we tried obtaining the complexes of hydroxypropyl-β-CD **(**HPβCD) with SCOs **2** in a 3:1 molar ratio, assuming that all 3 aromatic units of **2** were participating in complexation. The HPβCD-**2a** complexes were employed as model compounds in our solubility study.

**Figure 4:**
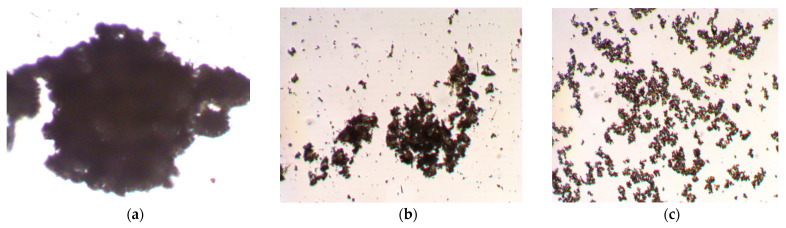
Influence of complexation with HPβCD on **2a** solubility (photomicrographs at ×10 magnification): suspensions of **2a** (**a**) and **HPβCD-2a** prepared by PM (**b**) and KM (**c**). C**_2a_** = 1 mg/mL, C_HPβCD_ = 12 mg/mL, pH 7.4.

Visually, complexation led to a significant increase in *S*. Although the bulk of **2a** remained undissolved, the HPβCD-**2a** complexes prepared via both PM and KM afforded white suspensions (Figure S1). According to our optical microscopy study, the crystal size significantly decreased for HPβCD-**2a** vs. free **2a** (Figure 4). The sample of **2a** contained large particles with arbitrary shapes (Figure 4a), whereas in the samples of **HPβCD-2a** prepared via PM (Figure 4b) and KM (Figure 4c), noticeably smaller uniform particles could be found.

**Figure 5:**
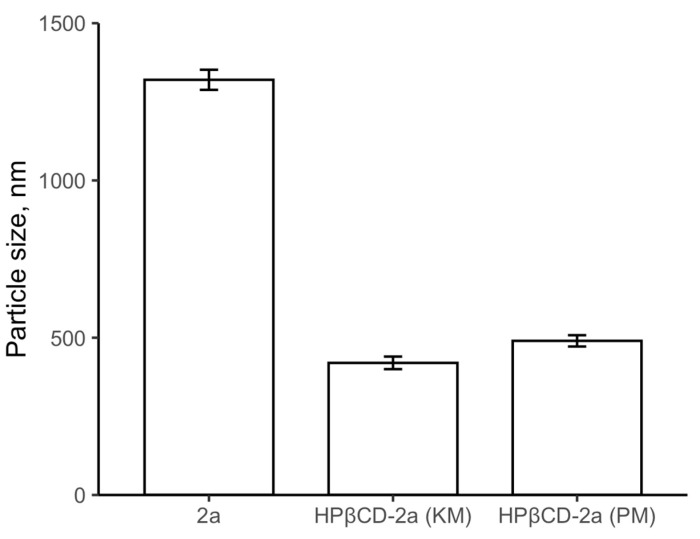
Size of particles in supernatants of **2a** and HPβCD-**2a** complex prepared by KM and PM. C**_2a_** =1 mg/mL, C**_HPβCD_** = 12 mg/mL, pH 7.4.

Next, the supernatants of these samples were analyzed via dynamic light scattering (DLS). Per this analysis, **2a** formed a homogenous solution with 1320 ± 32 nm particles (Figure 5, hydrodynamic diameter). The particles of the HPβCD-**2a** complex were significantly smaller: 490 ± 18 nm (PM) and 420 ± 20 nm (KM). This supports the increase in *S* upon complexation. The smaller size of particles provides a number of advantages: increased bioactivity, reduced side effects, and increased cell penetration.

Furthermore, the formation of the HPβCD-**2a** complex dramatically increased the intensity of the absorption maximum at ca. 265 nm, corresponding to **2a** in the UV spectra. The *S***_2a_** value (0.18 ± 0.02 mg/mL for free **2a**) increased up to 0.57 ± 0.05 mg/mL and 0.67 ± 0.04 mg/mL for **2a** in complexes prepared via PM and KM, respectively. As KM provided the highest *S***_2a_** value, we used this method for our further experiments.

### 3.4. Phase Solubility Studies

The phase solubility technique developed by Higuchi and Connons [24,25,26] was used to study the HPβCD-**2a** complex in order to elucidate how the concentration of HPβCD affected the guest compound solubility and to determine the host-guest molar ratio, as well as the value of *K*_c_, the binding constant.

**Figure 6:**
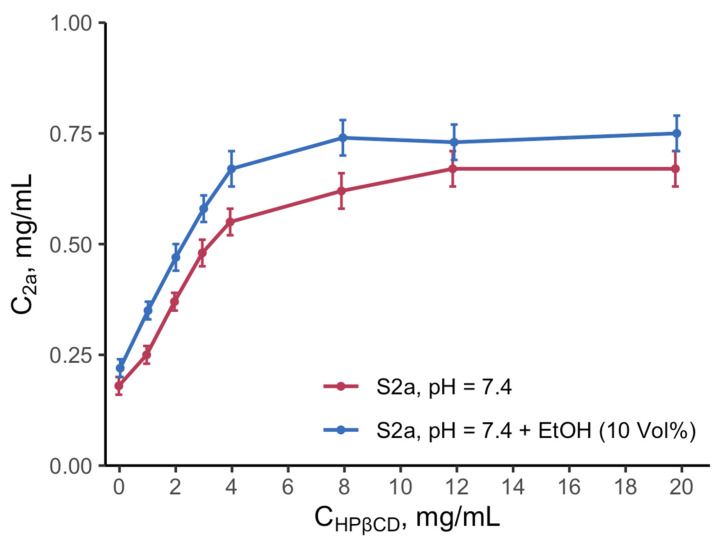
Phase-solubility profiles for HPβCD-**2a** complex in sodium phosphate buffer (pH 7.4) (red curve) and in sodium phosphate buffer (pH 7.4) with EtOH (10 vol%) (blue curve), C**_2a_** =1 mg/mL, shake-flask method.

According to the measured phase-solubility profile, at pH 7.4, *S***_2a_** linearly rises at low HPβCD concentrations until saturation (A_N_-type profile) (Figure 6, red curve). The decreased solubilization effect of CDs at higher concentrations can be attributed to the limited aqueous solubility of **2a**, the changes in viscosity and/or surface tension, or to self-association of CD molecules [24,42]. The highest *S***_2a_** value was determined at the HPβCD:**2a** molar ratio of 3:1, which might be explained by the saturation of all **2a** binding sites (all 3 aromatic fragments interact with CDs). We achieved an almost 4-fold increase of **2a** solubility (~0.2 mg/mL compared to 0.67 mg/mL).

The formation of ternary complexes might provide the enhancement of inclusion affinity [43]. We used ethanol as a third component that might also increase the *S*_2a_ value by the decrease in the medium polarity (Figure 6, blue curve). Indeed, ethanol (10 vol%) increased the solubility of **2a**, though the effect is not significant. The *K_c_* values were determined by analyzing the linear ranges of the isotherms for 1:1 stoichiometry of complexation [44,45]: *K*_c_ = 235 ± 8 M^−1^ (pH 7.4) and *K*_c_ = 277 ± 11 M^−1^ (pH 7.4 + 10 vol% EtOH). The determined *K*_c_ correspond to a range of values (ca. 50 ÷ 104 M^−1^) reported for the complexes with β-CDs with biologically active molecules [46,47,48,49].

### 3.5. Influence of Substituents at β-CDs on SCO Solubility

The nature of substituents in the CDs might also influence the solubility of the guest molecule. For example, a similar effect was revealed for β-CD complexes with fluoroquinolones, wherein the substituents in the β-CDs additionally interacted with guest molecules, increasing the efficiency of complexation [47].

To reveal the effect of the substituents in β-CDs on their complexation with **2a**, two additional common β-CDs were examined: methyl-β-CD (MβCD) and sulfobutyl ether β-CD (SBEβCD) [17,26]. In terms of overall substituent polarity, the examined β-CDs can be rated as follows: SBEβCD > HPβCD > MβCD. We found that varying β-CDs had no noticeable effect on the type of phase-solubility profiles or ranges of saturation. Nevertheless, differences in complexation efficiency at low β-CDs molar excess were detected: *K*_c_ = 235 ± 8 M^−1^ (HPβCD-**2a**), *K*_c_ = 294 ± 11 M^−1^ (MβCD-**2a**), *K*_c_ = 245 ± 9 M^−1^ (SBEβCD-**2a**).

As MβCD provided the highest solubility for **2a**, we also investigated the MβCD-**2b** complex. As the intrinsic *S***_2b_** value was found to be almost five times higher than that of *S***_2a_** (Table 1), we could expect a higher *K*_c_ value for MβCD-**2b** vs. MβCD-**2a**. Indeed, the *K*_c_ value of the MβCD-**2b** complex was 574 ± 12 M^−1^, ca. 2 times higher than that for MβCD-**2a**. Notably, for MβCD-**2b**, saturation was achieved at an MβCD:**2b** molar ratio of ca. 2:1, whereas for MβCD-**2a**, the ratio was ca. 3:1. As CDs mostly affect poorly soluble guest molecules, corresponding to Class II Drugs, according to the Biopharmaceutics Classification System [50], the limited effect of MβCD on the solubility of **2b** might be attributed to the high initial *S***_2b_** value.

**Figure 7:**
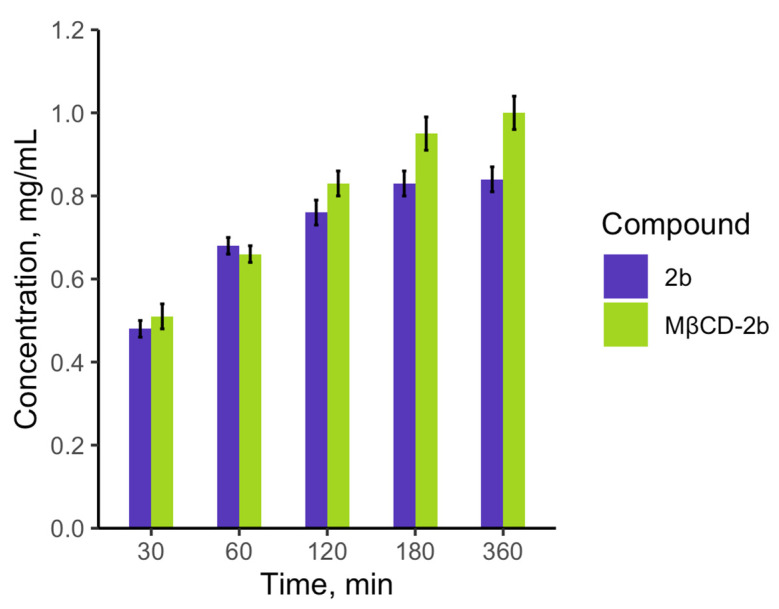
The dissolution rate of **2b** and MβCD-**2b**, sodium phosphate buffer (pH 7.4), shake-flask method.

Complex formation might change not only the solubility of the samples, but also the dissolution rate. The effect of stirring time on the equilibrium solubility is reasonable to be investigated during first 6 h [23]. Figure 7 demonstrates that MβCD pronouncedly increases the **2b** dissolution rate after 2 h of incubation. At 6 h, *S*_MβCD-2b_ is higher than *S*_2b_ on 15%. 

### 3.6. Characterization of Inclusion Complexes

#### 3.6.1. PXRD Analysis

**Figure 8:**
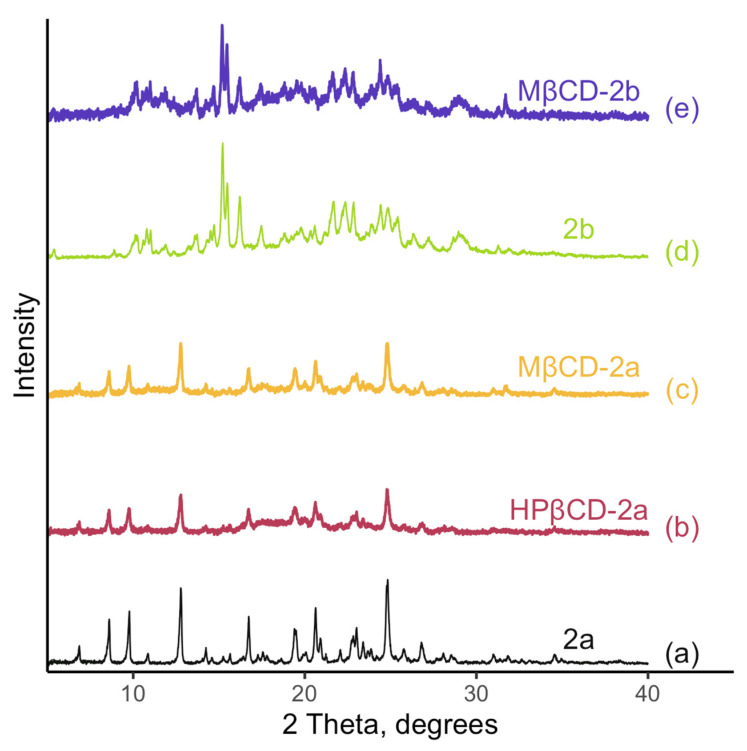
PXRD patterns for (**a**) **2a**; (**b**) HPβCD-**2a**; (**c**) MβCD-**2a**; (**d**) **2b**; (**e**) MβCD-**2b**. No signals were observed at 40−80°.

To determine the degree of crystallinity for SCOs **2a**,**b** and their complexes with β-CDs, we used powder X-ray diffraction (PXRD) [51,52]. Narrow intense peaks in the PXRD patterns of **2a** and **2b** point to the predominance of crystalline forms (Figure 8a,d). The PXRD patterns for MβCD mainly represent an amorphous state, agreeing with reported data [53]. The formation of the β-CD-SCO complex affects the PXRD pattern: a halo appears, corresponding to a decrease in the degree of crystallinity (Figure 8b,c,e). This agrees well with the data reported for other β-CD complexes [47].

The degree of crystallinity is a crucial factor in drug development. For instance, the amorphous intraconazole formulation exhibited significantly higher systemic bioavailability via pulmonary administration than the nanocrystalline drug form [54]. Therefore, the decrease in crystallinity for the β-CD-SCO complexes might improve the biological activity of SCO.

#### 3.6.2. FTIR Microscopy

The structures of the β-CD-SCO complexes were also studied via FTIR microscopy. This allowed us to detect changes in the microenvironments of the functional groups in molecules upon complexation. Moreover, macrostructure FTIR microscopy provides a pathway to determining the distribution of the guest molecules and the ratio of components in the complexes [55].

The main analytically significant absorption bands in the FTIR spectrum of HPβCD are located at 1220–950 cm^−1^ [56]. The most intense bands at 1032, 1083, and 1172 cm^–1^ correspond to the valence vibrations of C–O–C, C–H, and C–O–H groups, respectively.

**Figure 9:**
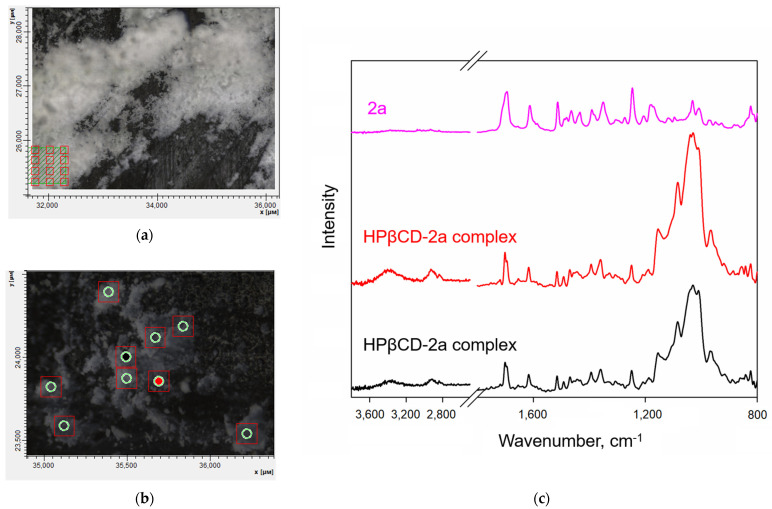
Microscopy photo of (**a**) **2a** and (**b**) HPβCD-**2a** complex. (**c**) FTIR spectra of **2a** (pink curve) and HPβCD-**2a** [red and black curves correspond to red and black areas circled in light-green in the photo (**b**)].

In our FTIR spectrum of **2a** (Figure 9c, Table 2), the broad peaks at 2935 and 2840 cm^–1^ correspond to C-H_Alk_ [57,58,59], while the intense peaks at 1697 and 1613 cm^–1^ correspond to amide I and II, respectively. The peaks in the 1430–1650 cm^–1^ range correspond to the C=C bonds in the aromatic rings.

All of these bands were observed in the FTIR spectra of the HPβCD-**2a** complex, as well (Figure 9c, Table 2). The intensity ratio for the peaks corresponding to HPβCD:**2a** was preserved for all the examined regions in the sample (Figure 9b, green circles), leading us to the conclusion that **2a** was uniformly distributed throughout the sample. The overall decrease in the intensity for the **2a** peaks might indicate complexation, as a similar effect was observed in the FTIR spectra of other β-CD-guest complexes [18].

In order to clarify the structure of HPβCD-**2a**, we took a closer look at the shifts in the positions of the initial **2a** bands (Table 2). The high-frequency shifts of the peaks at 1697, 1613, and 1650–1430 cm^−1^ uncovered that the N-C=O group and the aromatic rings were involved in complexation. As for the peaks corresponding to C-Cl (1031 cm^−1^) and C-O-C_Ar_ (1246 cm^−1^) in **2a**, the former did not change its position, whereas the latter underwent a high-frequency shift that could point to the PMB group being involved in complexation. We could not determine whether the CH_2_ groups participated in complexation because the C-HAlk vibrations region of 3050–2700 cm^−1^ is less informative due to the broadening and the decrease in the intensity of the bands. Thus, the FTIR results pointed to the formation of an HPβCD-**2a** inclusion complex, wherein the PMB group of **2a** was captured by the hydrophobic cavity of HPβCD, with the cyclopropane and *o*-chlorophenyl fragments sticking out.

#### 3.6.3. Two-Dimensional NMR Spectroscopy: ^1^H-^1^H ROESY Experiments

**Figure 10:**
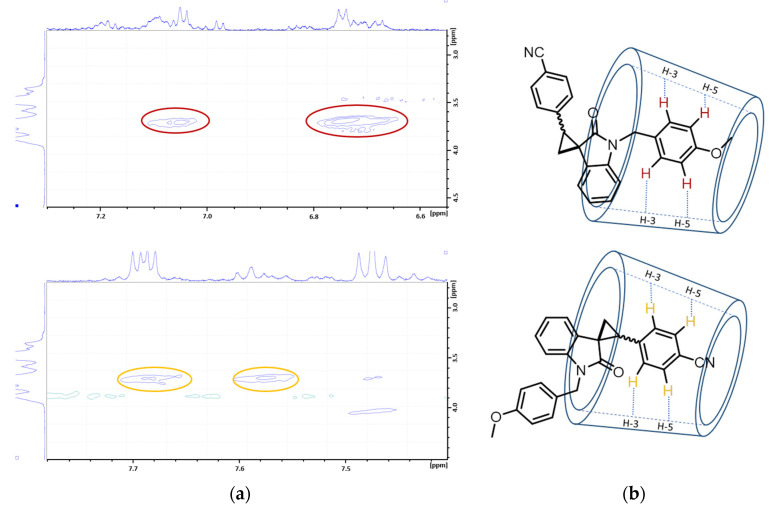
(**a**) Fragment of ^1^H-^1^H ROESY NMR spectrum of the HPβCD-**2b** complex; (**b**) possible inclusion modes of HPβCD-**2b**.

An ^1^H-^1^H ROESY NMR spectrum was detected for the HPβCD-**2b** complex to support the complexation hypothesis. The cross-peaks between the signals assigned to the H atoms of the PMB group in **2b** and the H-3 and H-5 atoms of HPβCD (Figure 10a, circled in red) indicated the capture of the PMB moiety by the hydrophobic cavity of HPβCD (Figure 10b). These results agree with those of the FTIR experiments quite nicely.

Meanwhile, the less intensive cross-peaks between the signals of the H atoms in the *p*-cyanophenyl ring and the H-3 and H-5 atoms of HPβCD were also detected (Figure 10a, circled in yellow). This pointed to the alternative possibility of complexation via insertion of the *p*-cyanophenyl group into the HPβCD cavity (Figure 10b).

#### 3.6.4. Molecular Modeling

As of this writing, no experimental structures have been reported for β-CD-SCO complexes. We carried out molecular modeling studies of the MβCD-**2a,b** complexes to determine the main β-CD-**2a,b** interactions and predict the stability of the corresponding complexes.

**Figure 11:**
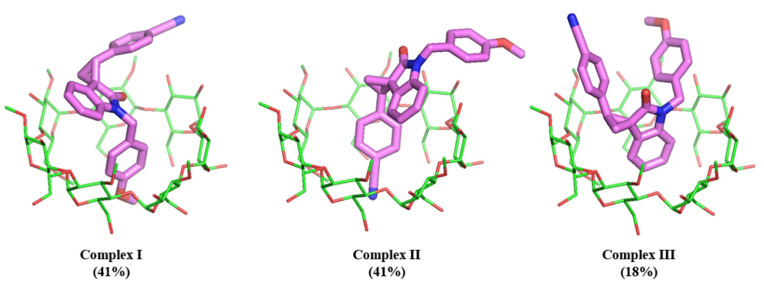
Three predicted MβCD-**2b** complex types.

First, all **2b** stereoisomers were docked into the MβCD cavity. Three possible binding modes (Type I-III) were predicted for the MβCD-**2b** complex (Figure 11).

The Type I and II conformations shared elongated geometries. On the one hand, this allowed them to enter the MβCD cavity with their PMB or *p*-cyanophenyl groups, respectively, resulting in hydrophobic interactions with the backbone atoms and the substituents in MβCD. On the other hand, the 3D configuration of the spiro-center in **2** oriented the substituent at the opposite side of the molecule to align it parallel to the MβCD backbone, forming numerous van der Waals contacts. Additionally, the CN and MeO groups were oriented towards the narrow rim of the MβCD torus and the water molecules. In turn, the water molecules were linked with the MeO groups in MβCD by fluctuating hydrogen bonds.

For the Type III conformation, **2b** adopted a U-shaped geometry, with the oxindole scaffold deeply buried in the MβCD cavity. Notably, the PMB and *p*-cyanophenyl moieties only formed a few hydrophobic contacts and hydrogen bonds with the water molecules of the wide MβCD rim.

MD simulations were performed to identify the favored binding mode, with the discovered Type I-III structures as initial ones.

**Figure 12:**
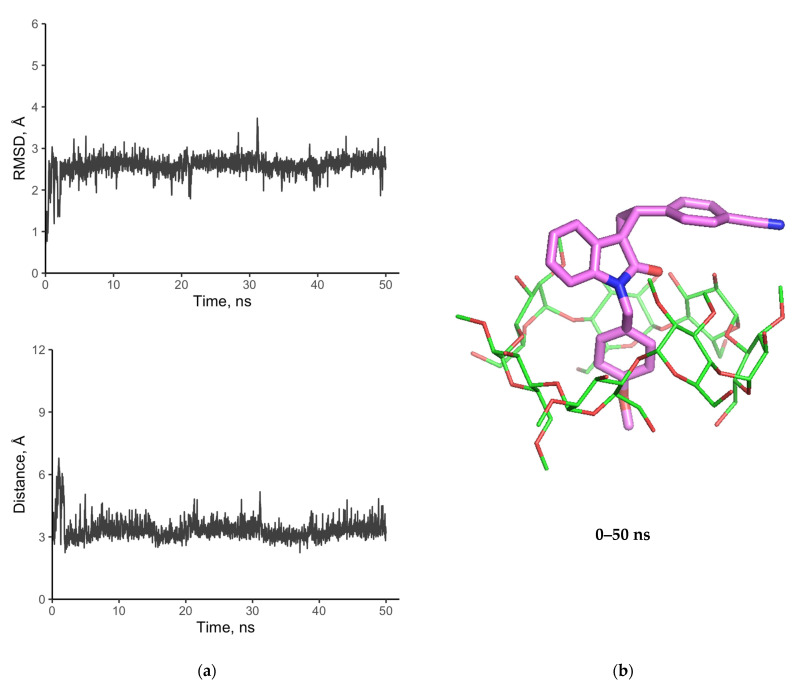
(**a**) Root-mean-square deviation (RMSD) and distance between the MβCD and **2b** centers of geometry (Type I complex). (**b**) Type I complex structure.

The root-mean-square deviation (RMSD) and the distance between the center of mass for **2b** and MβCD were monitored to quantitatively assess the structural fluctuations of each binding mode from the resulting trajectories. The Type I complex exhibited the most stable conformation throughout the entire dynamic trajectory (Figure 12), which agrees with the NMR results, supporting primary complexation via the interaction of β-CD with the PMB group in **2b**.

**Figure 13:**
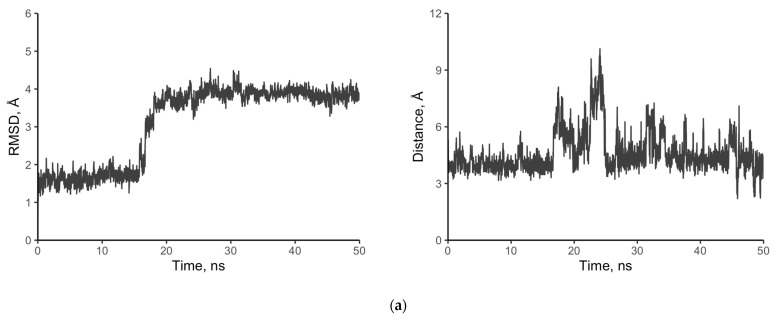

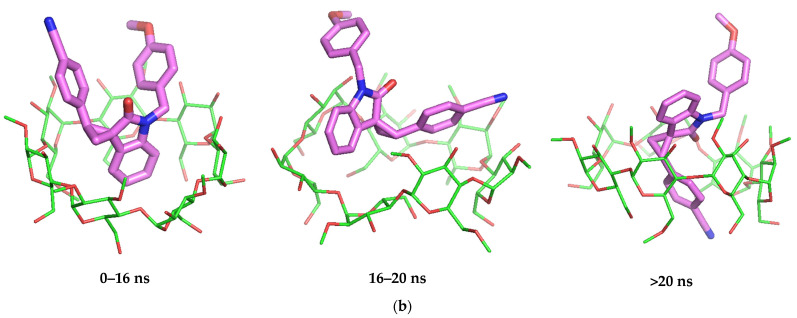
(**a**) Root-mean-square deviation (RMSD) and distance between the MβCD and **2b** centers of geometry (Type III complex). (**b**) Type III complex structure transformation into Type II geometry.

At the same time, the Type III conformation with the highest docking score was stable for the first 16 ns of the molecular dynamics trajectory. In our simulation, the complex dissociated between 16 and 20 ns (Figure 13a) to form a new stable Type II geometry, with the *p*-cyanophenyl group deep in the MβCD cavity (Figure 13b).

**Figure 14:**
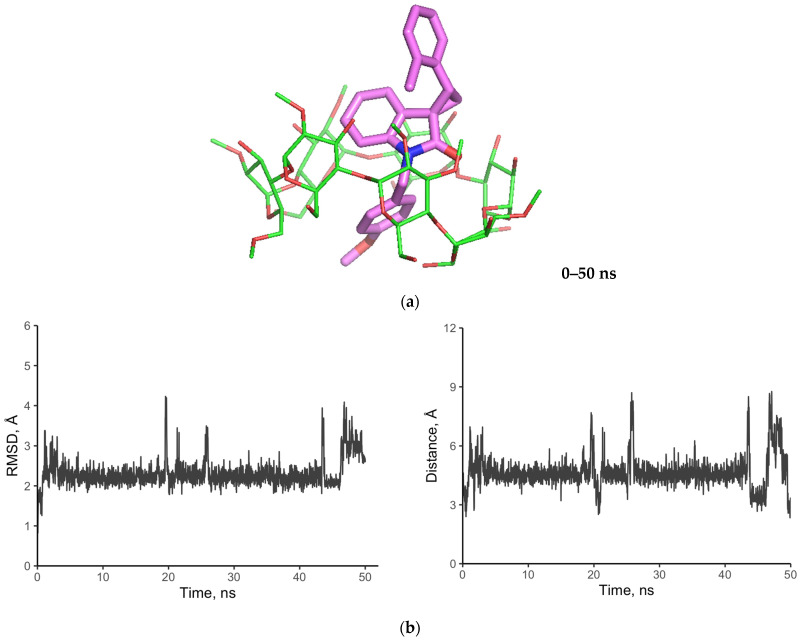
(**a**) Type I complex conformation; (**b**) MβCD and **2a** heavy atoms positions root-mean-square deviation (RMSD) and distance between the MβCD and **2a** centers of geometry (Type I complex).

Molecular docking also revealed that the **2a** isomers bind MβCD similarly to the **2b** isomers, forming three types of complexes via the MβCD cavity, capturing one of the three available aromatic fragments. The elongated conformations of the **2a** isomers readily formed complexes, wherein the PMB group was deeply buried in the MβCD cavity (Figure 14a, complex I). This binding mode is in accordance with the FTIR spectroscopic results. The molecular dynamics trajectory for the Type I complex did not show any significant perturbations for the ligand conformation (Figure 14b), possibly pointing to the stability of this type.

### 3.7. Biological Activity of SCOs In Vitro 

We tested the potential in vitro activity of **2a,b** SCOs against the gram-negative *E.coli* ATCC 25922 strain.

**Figure 15:**
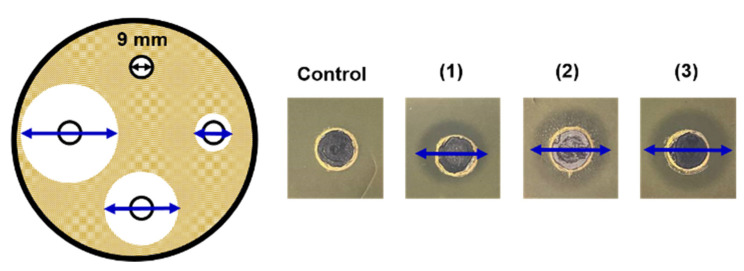
The scheme of the agar well diffusion method. The inhibition zones on Petri dishes appeared after treatment of (1) **2b** 5 μg/mL; (2) **2b** 7 μg/mL; (3) HPβCD-**2b** 10 μg/mL; pH 7.4 (0.01 M sodium phosphate buffer), 37 °C, 24 h of incubation. Blue arrows demonstrate the diameter of the inhibition zone.

The minimum inhibition concentration (MIC) was determined via the agar well diffusion method (Figure 15), a fast and robust technique [27,51]. Both **2a** and **2b** inhibited bacteria growth (MIC_2a_~1000 μg/mL; MIC_2b_~3 μg/mL) (Table 3), with dose dependency characterizing their antibacterial effect. The significant difference between the MIC values might be associated with differences between the *S***_2a_** and *S***_2b_** values.

As expected, both HPβCD and MβCD proved to be non-toxic and biodegradable [11,28]. As CDs can decrease the MIC values [51,60], we studied the antibacterial activity of **2a** and **2b** complexes with βCDs. Both HPβCD and MβCD decreased the MIC**_2a_** values sevenfold. Nevertheless, the MIC**_2b_** values remained the same, despite complexation. Apparently, this is related to a significant increase in *S***_2a_** via complexation with βCDs, whereas complexation affects *S***_2b_** only slightly.

## 4. Conclusions

In conclusion, the strategy of increasing the water solubility of poorly soluble small molecules via complexation with CDs was successfully applied for a promising class of organic molecules, spiro[cyclopropane-1,3′-oxindoles], which are currently under active development in preclinical and clinical trials.

The complexation of spiro[cyclopropane-1,3′-oxindoles] with different β-CDs was first evaluated by phase solubility and optical microscopy studies. The PXRD analysis was also conducted, confirming the crystal size reduction. These results indicate a significant influence of β-CDs on the biopharmaceutical properties of synthesized SCO.

Then, the binding patterns of the observed SCO-βCD interaction were established by FTIR, 2D NMR, and molecular modeling experiments, proving the entrapment of SCO aromatic rings into the hydrophobic cavity of CD. The revealed data are in good agreement in all the cases, providing the most probable mechanism of SCO-βCD complexation.

Furthermore, it has been evidenced that SCO-βCDs were capable of inhibiting bacterial growth. In addition, complexation allowed a significant MIC decrease in the case of SCO with low intrinsic solubility and subsequently revealed an antibacterial effect. These facts elucidate that SCOs are perspective antibacterial agents, and their complexation with CDs is a promising strategy to enhance the water solubility, biological activity, and biopharmaceutical properties of spirooxindole derivatives.

## Figures and Tables

**Table 1:** Intrinsic solubilities (S) of SCOs **2**.

2	Ar	*S*^a^, mg/mL
**a**	2-ClC_6_H_4_	0.18 ± 0.02
**b**	4-NCC_6_H_4_	0.84 ± 0.03
**c**	4-*t*-BuC_6_H_4_	0.11 ± 0.02
**d**	3,4-(MeO)_2_C_6_H_3_	0.27 ± 0.03

^a^ Suspensions of **2** (1 mg/mL) in sodium phosphate buffer (pH 7.4), shake-flask method.

**Table 2:** Peaks in FTIR spectra of **2a** and HPβCD-**2a**, cm^−1^.

	2a	HPβCD-2a
C-H_Alk_	2935 ± 0.5	2935 ± 0.5
	2840 ± 0.5	2840 ± 0.5
Amide I (C=O)	1697 ± 0.5	1701 ± 0.5
Amide II (N-C=O)	1613 ± 0.5	1617 ± 0.5
C_Ar_-H	1481 ± 0.5	-
C-O-C_Ar_	1246 ± 0.5	1248 ± 0.5
C_Ar_-Cl	1031 ± 0.5	1030 ± 0.5

**Table 3:** The MIC values of different samples, pH 7.4 (0.01 M PBS), 37 °C.

MIC, μg/mL	CD	2a	2b
Without CD		1000 ± 35	3.2 ± 0.3
HPβCD	−	150 ± 20	3.0 ± 0.4
MβCD	−	140 ± 23	3.4 ± 0.4

## Data Availability

All data generated or analyzed during this study are included in this published article and its Supplementary Information.

## Supplementary Materials

The following supplementary materials can be downloaded at: https://www.mdpi.com/article/10.3390/pharmaceutics15010228/s1, Figure S1: Influence of HPβCD on 2a solubility; Figure S2: RMSD and Distance between the centers of geometry for MβCD-2 (all complexes are shown); Table S1: Molecular formula strings, IUPAC names and docking scores.
