# Supplementary material for: The Solubility Studies and the Complexation Mechanism Investigations of Biologically Active Spiro[cyclopropane-1,3′-oxindoles] with β-Cyclodextrins
Source: Pharmaceutics. 2023 Jan 9;15(1):228. doi: 10.3390/pharmaceutics15010228 (PMC9861668; doi:10.3390/pharmaceutics15010228)

Supporting Information

The Solubility Studies and the Complexation Mechanism Investigations of Biologically Active Spiro[cyclopropane-1,3′-oxindoles] with β-Cyclodextrins

Anna A. Kravtsova [1,2], Anna A. Skuredina [1], Alexander S. Malyshev [2,3], Irina M. Le-Deygen [1,*], Elena V. Kudryashova [1] and Ekaterina M. Budynina [1]

[1] Department of Chemistry, Lomonosov Moscow State University, 119991 Moscow, Russia
[2] Faculty of Medicine, Lomonosov Moscow State University, 119991 Moscow, Russia
[3] Dukhov Research Institute of Automatics (VNIIA), 127030 Moscow, Russia

Table of Contents

Figure S1. Influence of HPβCD on **2a** solubility.	S2
Table S1. Molecular formula strings, IUPAC names and docking scores.	S3
Figure S2. RMSD and Distance between the centers of geometry for **MβCD-2** (all complexes are shown).	S4
Analytical data for the synthetized compounds	S5
^{1}H and ^{13}C NMR spectra.	S7

(a) (b) (c) (d)

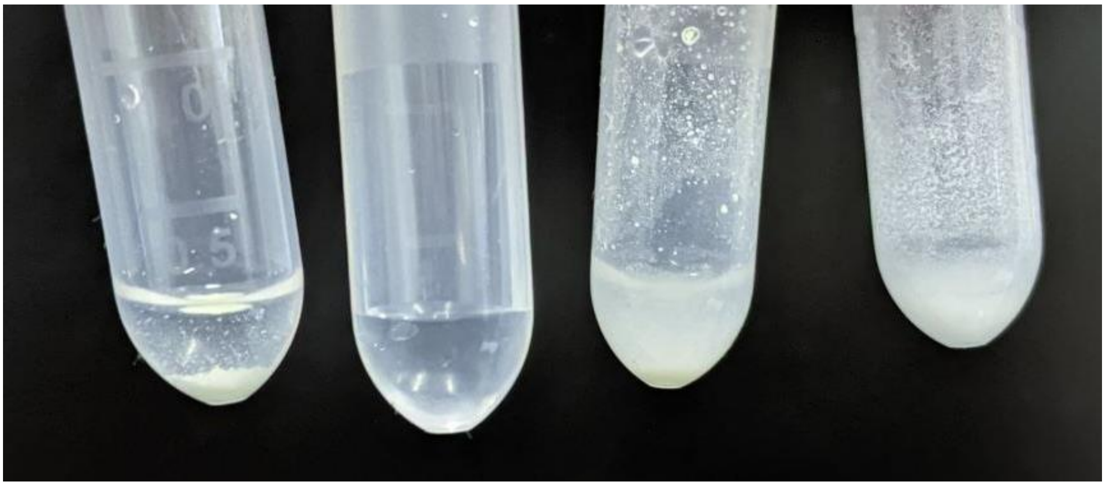

Figure S3. Influence of HPβCD on **2a** solubility. The suspension of **2a** (a), solution of **HPβCD** (b)**, HPβCD-2a** prepared by PM (c) and KM (d) methods.

Table S2. Molecular formula strings, IUPAC names and docking scores.

ID	SMILES	IUPAC Name	Docking Score
2a_1	COc1ccc(CN(c2c([C@@]34[C@H](c5ccccc5Cl)C4)cccc2)C3=O)cc1	(1R,2R)-2-(2-chlorophenyl)-1'-(4-methoxybenzyl)spiro[cyclopropane-1,3'-indolin]-2'-one	-4.822
2a_2	COc1ccc(CN(c2c([C@]34[C@H](c5ccccc5Cl)C4)cccc2)C3=O)cc1	(1S,2R)-2-(2-chlorophenyl)-1'-(4-methoxybenzyl)spiro[cyclopropane-1,3'-indolin]-2'-one	-5.035
2a_3	COc1ccc(CN(c2c([C@@]34[C@@H](c5ccccc5Cl)C4)cccc2)C3=O)cc1	(1R,2S)-2-(2-chlorophenyl)-1'-(4-methoxybenzyl)spiro[cyclopropane-1,3'-indolin]-2'-one	-5.081
2a_4	COc1ccc(CN(c2c([C@]34[C@@H](c5ccccc5Cl)C4)cccc2)C3=O)cc1	(1S,2S)-2-(2-chlorophenyl)-1'-(4-methoxybenzyl)spiro[cyclopropane-1,3'-indolin]-2'-one	-4.920
2b_1	N#Cc1ccc([C@@H]2C[C@]23c4ccccc4N(Cc5ccc(OC)cc5)C3=O)cc1	4-((1R,2S)-1'-(4-methoxybenzyl)-2'-oxospiro[cyclopropane-1,3'-indolin]-2-yl)benzonitrile	-4.632
2b_2	N#Cc1ccc([C@@H]2C[C@@]23c4ccccc4N(Cc5ccc(OC)cc5)C3=O)cc1	4-((1S,2S)-1'-(4-methoxybenzyl)-2'-oxospiro[cyclopropane-1,3'-indolin]-2-yl)benzonitrile	-5.101
2b_3	N#Cc1ccc([C@H]2C[C@]23c4ccccc4N(Cc5ccc(OC)cc5)C3=O)cc1	4-((1R,2R)-1'-(4-methoxybenzyl)-2'-oxospiro[cyclopropane-1,3'-indolin]-2-yl)benzonitrile	-4.997
2b_4	N#Cc1ccc([C@H]2C[C@@]23c4ccccc4N(Cc5ccc(OC)cc5)C3=O)cc1	4-((1S,2R)-1'-(4-methoxybenzyl)-2'-oxospiro[cyclopropane-1,3'-indolin]-2-yl)benzonitrile	-4.713

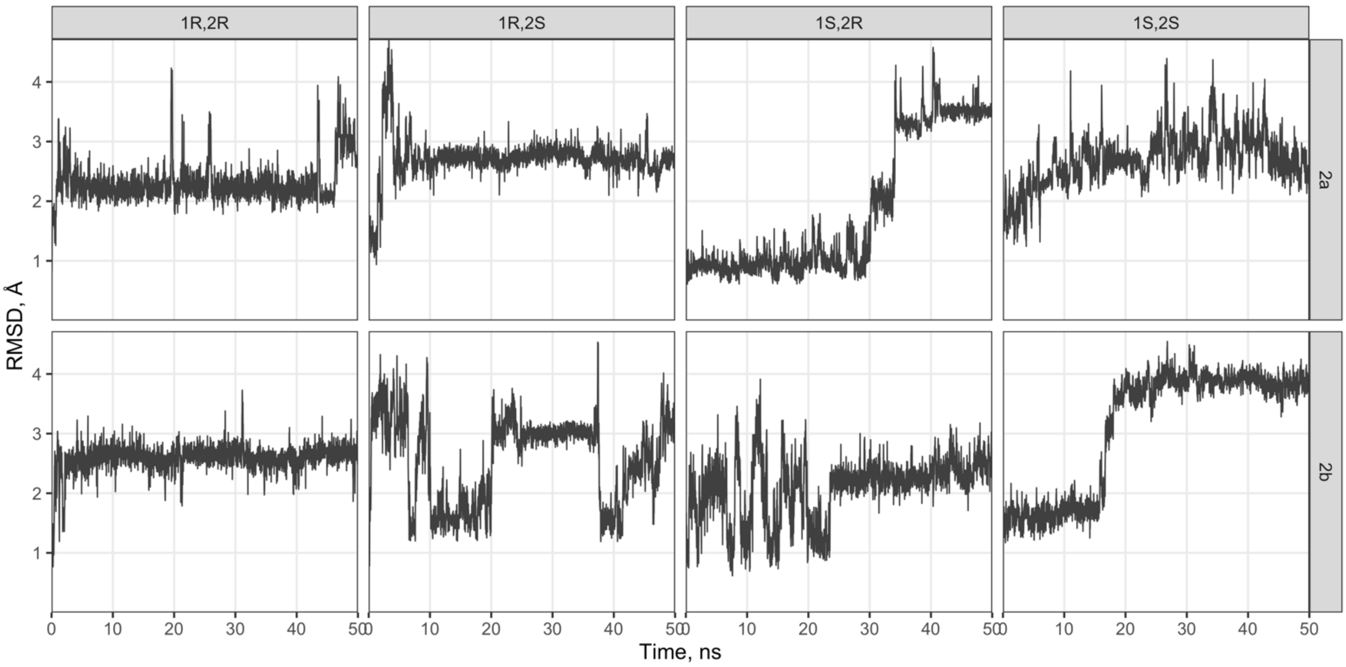

(a)

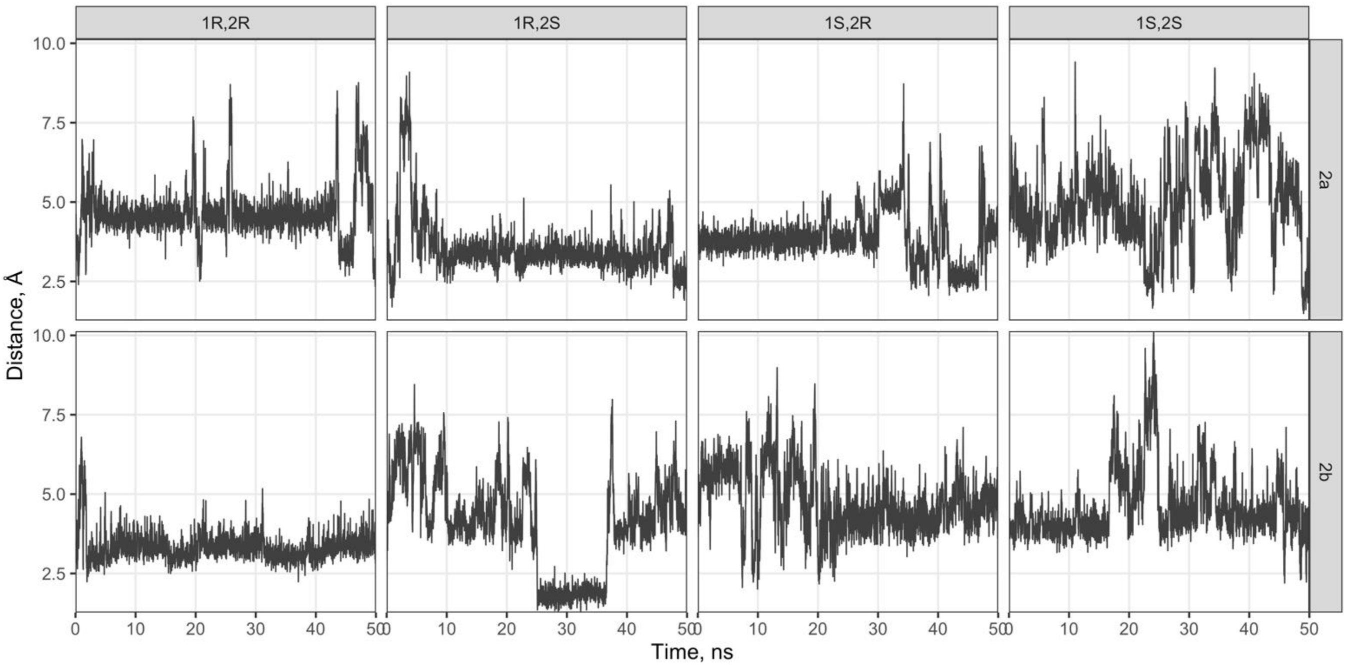

(b)

Figure S4. (a) Root mean square deviation (RMSD) and (b) Distance between the centers of geometry for **MβCD-2** (all complexes are shown).

3-(2-Chlorobenzylidene)-1-(4-methoxybenzyl)indolin-2-one (1a).

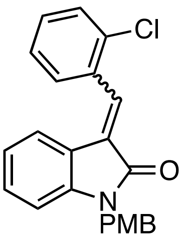

1a was obtained from 1-methylindolin-2-one (5.4 g, 21.3 mmol) and 2-chlorobenzaldehyde (2.6 mL, 23.5 mmol). Reaction time 2 h. Yield 4.8 g (60%), yellow oil, E:Z = 85:15. R_f = 0.50 (E), 0.65 (Z) (ethyl acetate/petroleum ether; 1:3).

E-1a: ^{1}H NMR ($CDCl_3$, 600 MHz) δ = 3.77 (s, 3H, CH_3O), 4.94 (s, 2H, CH_2N), 6.77 (br.d, ^3J = 7.9 Hz, 1H, Ar), 6.80–6.83 (m, 1H, Ar), 6.87 (d, ^3J = 8.8 Hz, 2H, Ar), 7.15–7.18 (m, 1H, Ar), 7.33 (d, ^3J = 8.8 Hz, 2H, Ar), 7.34–7.36 (m, 2H, Ar), 7.37–7.39 (m, 1H, Ar), 7.51 (dd, ^3J = 7.9, ^4J = 1.3 Hz, 1H, Ar), 7.72–7.73 (m, 1H, Ar), 7.98 (s, 1H, CH=).

^{13}C NMR ($CDCl_3$, 150 MHz) δ = 43.0 (CH_2N), 55.0 (CH_3O), 109.1 (CH), 114.0 (2×CH), 120.7 (C), 121.7 (CH), 122.8 (CH), 126.5 (CH), 127.8 (C), 128.4 (C), 128.6 (2×CH), 129.8 (CH), 129.9 (CH), 130.0 (CH), 130.5 (CH), 133.4 (C), 133.5 (CH), 134.2 (C), 143.4 (C), 158.9 (C), 167.7 (CO).

HRMS (ESI) m/z $[M + H]^+$ calcd for $C_{23}H_{19}ClNO_2^+$ 376.1099, found 376.1105.

3-(3,4-Dimethoxybenzylidene)-1-(4-methoxybenzyl)indolin-2-one (1d).

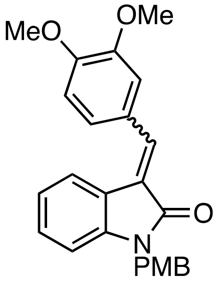

1d was obtained from 1-methylindolin-2-one (5.40 g, 21.3 mmol) and 3,4-dimethoxybenzaldehyde (3.90 g, 23.5 mmol). Reaction time 2 h. Yield 5.3 g (62%); yellow oil, E:Z = 84:16; R_f = 0.49 (E), 0.56 (Z) (ethyl acetate/petroleum ether; 3:2).

E-1d: ^{1}H NMR ($CDCl_3$, 600 MHz) δ = 3.78 (s, 3H, CH_3O), 3.91 (s, 3H, CH_3O), 3.97 (s, 3H, CH3O), 4.95 (s, 2H, CH2N), 6.77 (br.d, ^{3}J = 7.8 Hz, 1H, Ar), 6.86 (d, ^{3}J = 8.7 Hz, 2H, Ar), 6.87–6.89 (m, 1H, Ar), 6.97 (d, ^{3}J = 8.3 Hz, 1H, Ar), 7.15–7.18 (m, 1H, Ar), 7.25 (m, 1H, Ar), 7.29–7.30 (m, 2H, Ar), 7.32–7.33 (m, 1H, Ar), 7.80 (br.d, ^{3}J = 7.7 Hz, 1H, Ar), 7.87 (s, 1H, CH=).

^{13}C NMR ($CDCl_3$, 150 MHz) δ = 43.2 (CH_2N), 55.2 (CH_3O), 56.0 (2×CH_3O), 109.2 (CH), 111.0 (CH), 112.3 (CH), 114.1 (2×CH), 121.6 (CH), 122.5 (CH), 123.4 (CH), 125.4 (C), 127.5 (C), 128.2 (C), 128.6 (C), 128.7 (2×CH), 129.3 (CH), 137.8 (CH), 143.2 (C), 148.8 (C), 150.4 (C), 159.0 (C), 168.8 (CO).

HRMS (ESI) m/z $[M + H]^+$ calcd for $C_{25}H_{24}NO_4^+$ 402.1700, found 402.1689.

2-(2-Chlorophenyl)-1'-(4-methoxybenzyl)spiro[cyclopropane-1,3'-indolin]-2'-one (2a).

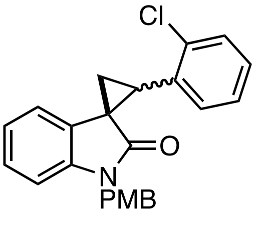

2a was obtained from **1a** (1.25 g, 3.33 mmol) according to the general procedure. Reaction time 3 h. Yield 1.19 g (92%); yellow oil. dr A:B = 71:29, R_f =0.56 (A), 0.46 (B) (ethyl acetate – petroleum ether; 1:2).

^{1}H NMR ($CDCl_3$, 600 MHz) δ = 2.07 (dd, ^2J = 4.8, ^3J = 8.1 Hz, 1H, CH_2, **A**), 2.13 (dd, ^2J = 4.9, ^3J = 8.8 Hz, 1H, CH_2, **B**), 2.36 (dd, ^2J = 4.8, ^3J = 8.9 Hz, 1H, CH_2, **A**), 2.43 (dd, ^2J = 4.9, ^3J = 8.5 Hz, 1H, CH_2, **B**), 3.14–3.17 (m, 1H, CH, **B**), 3.28–3.31 (m, 1H, CH, **A**), 3.78 (s, 3H, CH_3O, **B**), 3.79 (s, 3H, CH_3O, **A**), 4.74 (d, ^2J = 15.5, 1H, CH_2N, **B**), 4.84 (d, ^2J = 15.5 Hz, 1H, CH_2N, **A**), 4.93 (d, ^2J = 15.5, 1H, CH_2N, **B**), 5.19 (d, ^2J = 15.5 Hz, 1H, CH_2N, **A**), 5.87 (br.d, ^3J = 7.6, 1H, Ar, **A**), 6.61–6.63 (m, 1H, Ar, **A**), 6.78 (d, ^2J = 7.8 Hz, 1H, Ar, **A**), 6.81 (br.d, ^2J = 7.8 Hz, 1H, Ar, **B**), 6.83 (br.d, ^2J = 8.4 Hz, 2H, Ar, **B**), 6.87 (br.d,

^{2}J = 8.5 Hz, 2H, Ar, **A**), 7.01–7.04 (m, 1H, Ar, **A**), 7.05–7.07 (m, 2H, Ar, **B**), 7.17–7.20 (m, 1H, Ar, **B**), 7.22 (br.d, ^{2}J = 8.4 Hz, 2H, Ar, **B**), 7.23–7.24 (m, 2H, Ar, **A**), 7.25–7.27 (m, 1H, Ar, **B**), 7.31 (br.d, ^{2}J = 8.5 Hz, 2H, Ar, **A**), 7.33–7.37 (m, 1H + 2H, Ar, **A + B**), 7.48 (d, ^{2}J = 7.8 Hz, 1H, Ar, **A**), 7.50 (d, ^{2}J = 7.8 Hz, 1H, Ar, **B**).

A: ^{13}C NMR ($CDCl_3$, 150 MHz) δ = 21.6 (CH_2), 33.1 (C), 35.0 (CH), 43.5 (CH_2N), 55.2 (CH_3O), 108.77 (CH), 114.0 (2×CH, Ar), 119.3 (CH, Ar), 121.3 (CH, Ar), 126.6 (2×CH, Ar), 127.0 (C, Ar), 128.2 (C, Ar), 128.5 (2×CH, Ar), 128.8 (CH, Ar), 129.3 (CH, Ar), 130.1 (CH, Ar), 133.8 (C, Ar), 136.9 (C, Ar), 143.0 (C, Ar), 158.9 (C, Ar), 176.1 (CO).

B: ^{13}C NMR ($CDCl_3$, 150 MHz) δ = 22.3 (CH_2), 33.3 (C), 35.9 (CH), 43.3 (CH_2N), 55.2 (CH_3O), 108.83 (CH), 113.9 (2×CH, Ar), 118.2 (CH, Ar), 121.8 (CH, Ar), 126.4 (CH, Ar), 126.8 (CH, Ar), 128.3 (C, Ar), 128.6 (CH, Ar), 128.7 (2×CH, Ar), 128.8 (CH, Ar), 130.3 (C, Ar), 130.8 (CH, Ar), 133.4 (C, Ar), 135.7 (C, Ar), 142.7 (C, Ar), 158.8 (C, Ar), 173.7 (CO).

HRMS (ESI) m/z $[M + H]^+$ calcd for $C_{24}H_{21}ClNO_2^+$ 390.1255, found 390.1257.

2-(3,4-Dimethoxyphenyl)-1'-(4-methoxybenzyl)spiro[cyclopropane-1,3'-indolin]-2'-one (2d).

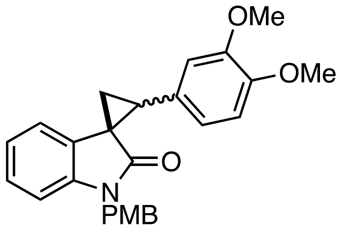

2d was obtained from **1d** (1.25 g, 3.12 mmol) according to the general procedure. Reaction time 2 h. Yield 1.11 g (86%); yellowish oil; dr A:B = 70:30; R_f =0.48 (A), 0.38 (B) (ethyl acetate – petroleum ether; 1:2).

A: ^{1}H NMR ($CDCl_3$, 600 MHz) δ = 1.99 (dd, ^{2}J = 4.4, ^{3}J = 7.9 Hz, 1H, CH_2), 2.24 (dd, ^{2}J = 4.4, ^{3}J = 9.2 Hz, 1H, CH_2), 3.34–3.37 (m, 1H, CH), 3.76 (s, 3H, CH_3O), 3.78 (s, 3H, CH_3O), 3.88 (s, 3H, CH_3O), 4.90 (d, ^{2}J = 15.4, 1H, CH_2N), 5.05 (d, ^{2}J = 15.4 Hz, 1H, CH_2N), 6.05 (d, ^{3}J = 7.5 Hz, 1H, Ar), 6.66 (m, 1H, Ar), 6.67–6.69 (m, 1H, Ar), 6.80–6.82 (m, 3H, Ar), 6.86–6.88 (m, 2H, Ar), 7.04–7.07 (m, 1H, Ar,), 7.29–7.30 (m, 2H, Ar).

A: ^{13}C NMR ($CDCl_3$, 150 MHz) δ = 23.0 (CH_2), 33.3 (C), 35.8 (CH), 43.6 (CH_2N), 55.2(CH_3O), 55.80 (CH_3O), 55.84 (CH_3O), 108.6 (CH), 110.9 (CH), 113.3 (CH, Ar), 114.1 (2×CH, Ar), 120.8 (CH, Ar), 121.4 (CH, Ar), 121.9 (CH, Ar), 126.3 (CH, Ar), 127.5 (C, Ar), 127.6 (C, Ar), 128.4 (C, Ar), 128.7 (2×CH, Ar), 142.8 (C, Ar), 148.3 (C, Ar), 148.7 (C, Ar), 159.0 (C, Ar), 176.4 (CO).

HRMS (ESI) m/z $[M + H]^+$ calcd for $C_{26}H_{26}NO_4^+$ 416.1856, found 416.1863.

3-(2-Chlorobenzylidene)-1-(4-methoxybenzyl)indolin-2-one (1a)

^{1}H NMR ($CDCl_3$, 600 MHz)

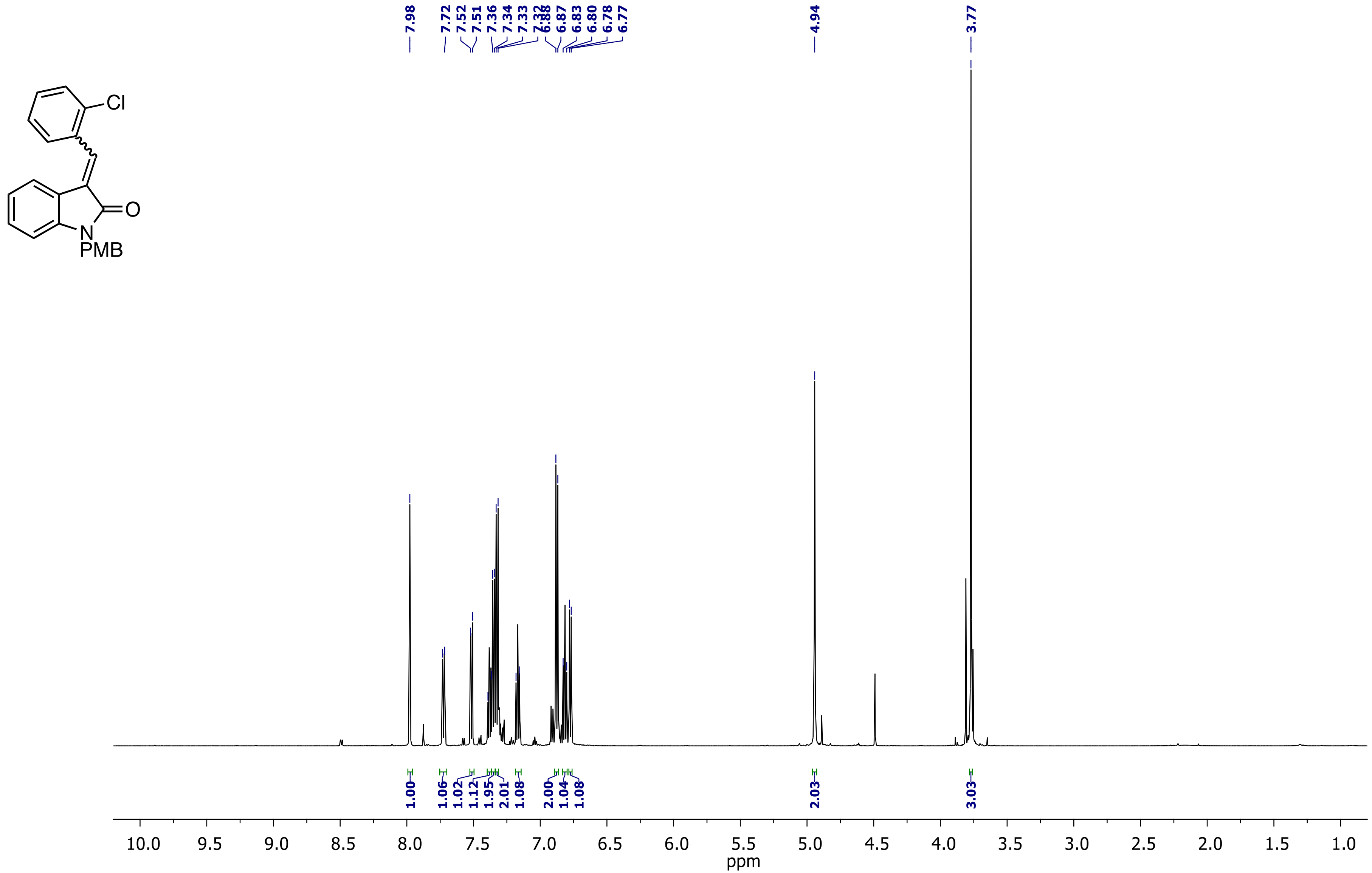

3-(2-Chlorobenzylidene)-1-(4-methoxybenzyl)indolin-2-one (1a)

^{13}C NMR (CDCl$_3$, 150 MHz)

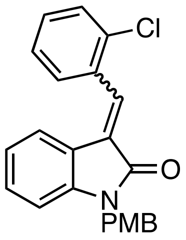

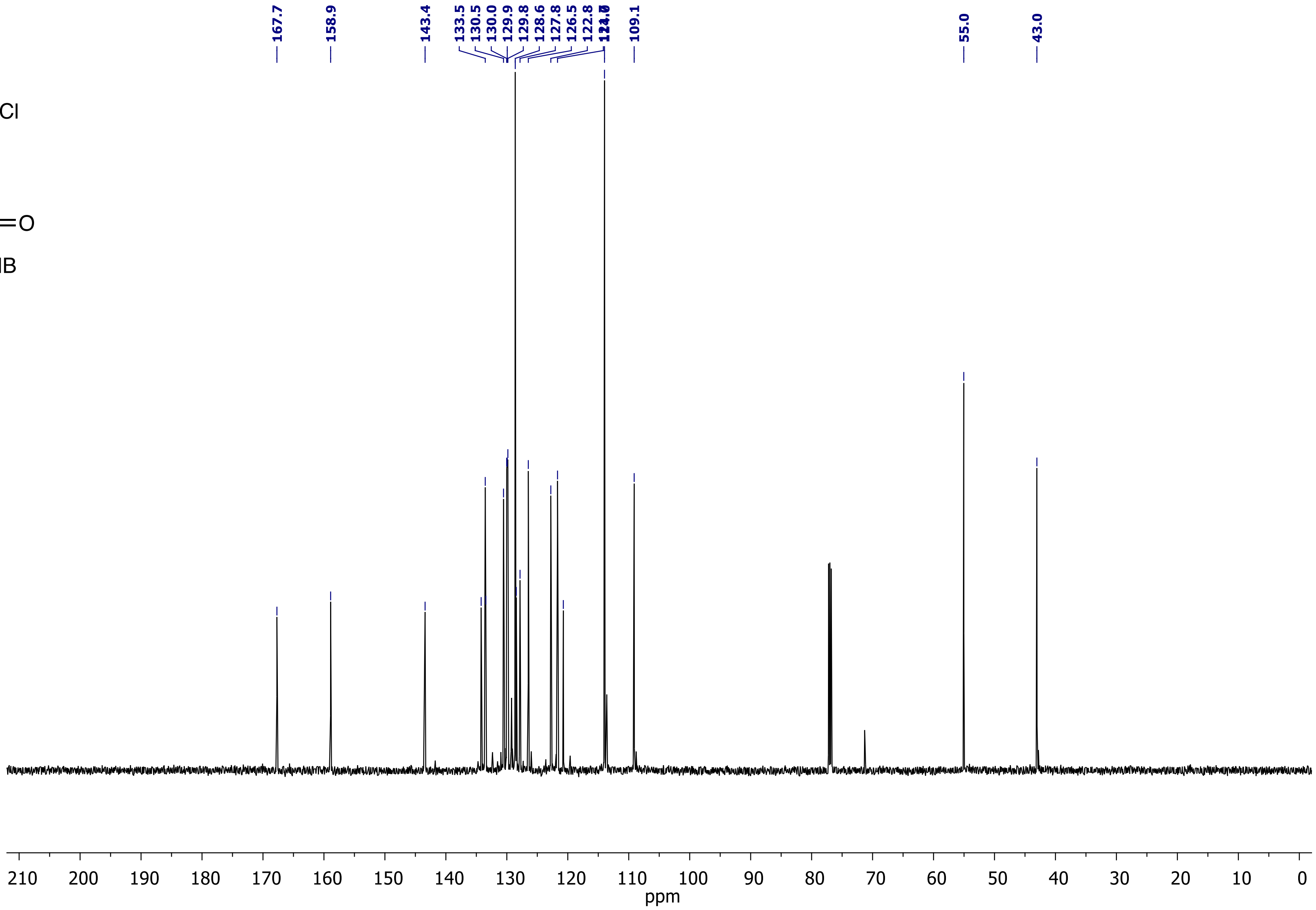

3-(3,4-Dimethoxybenzylidene)-1-(4-methoxybenzyl)indolin-2-one (1d)

^{1}H NMR ($CDCl_3$, 600 MHz)

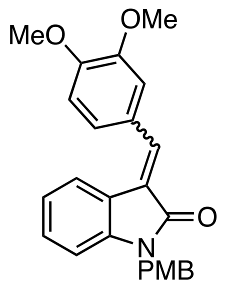

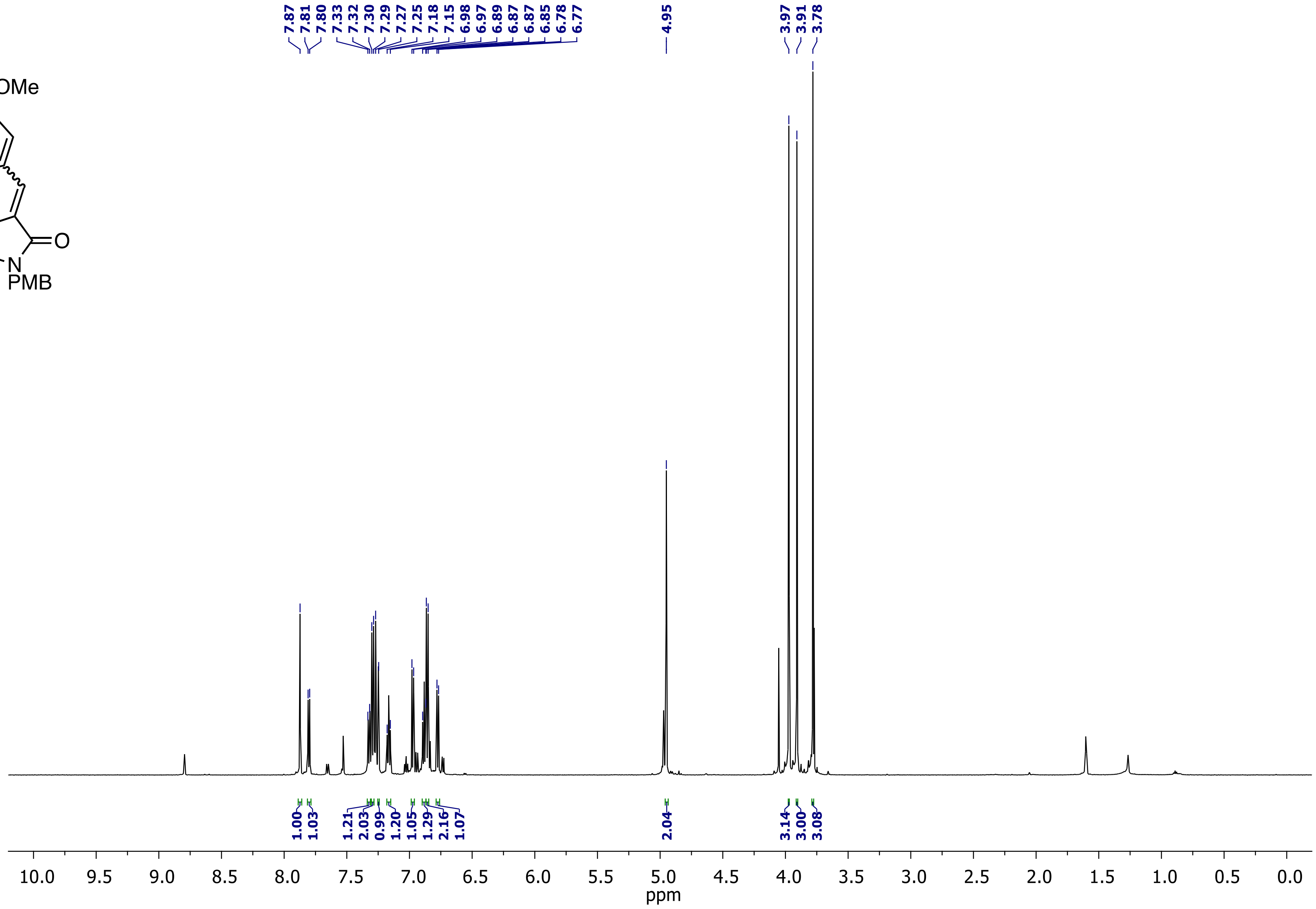

3-(3,4-Dimethoxybenzylidene)-1-(4-methoxybenzyl)indolin-2-one (1d)

^{13}C NMR ($CDCl_3$, 150 MHz)

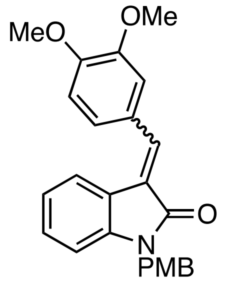

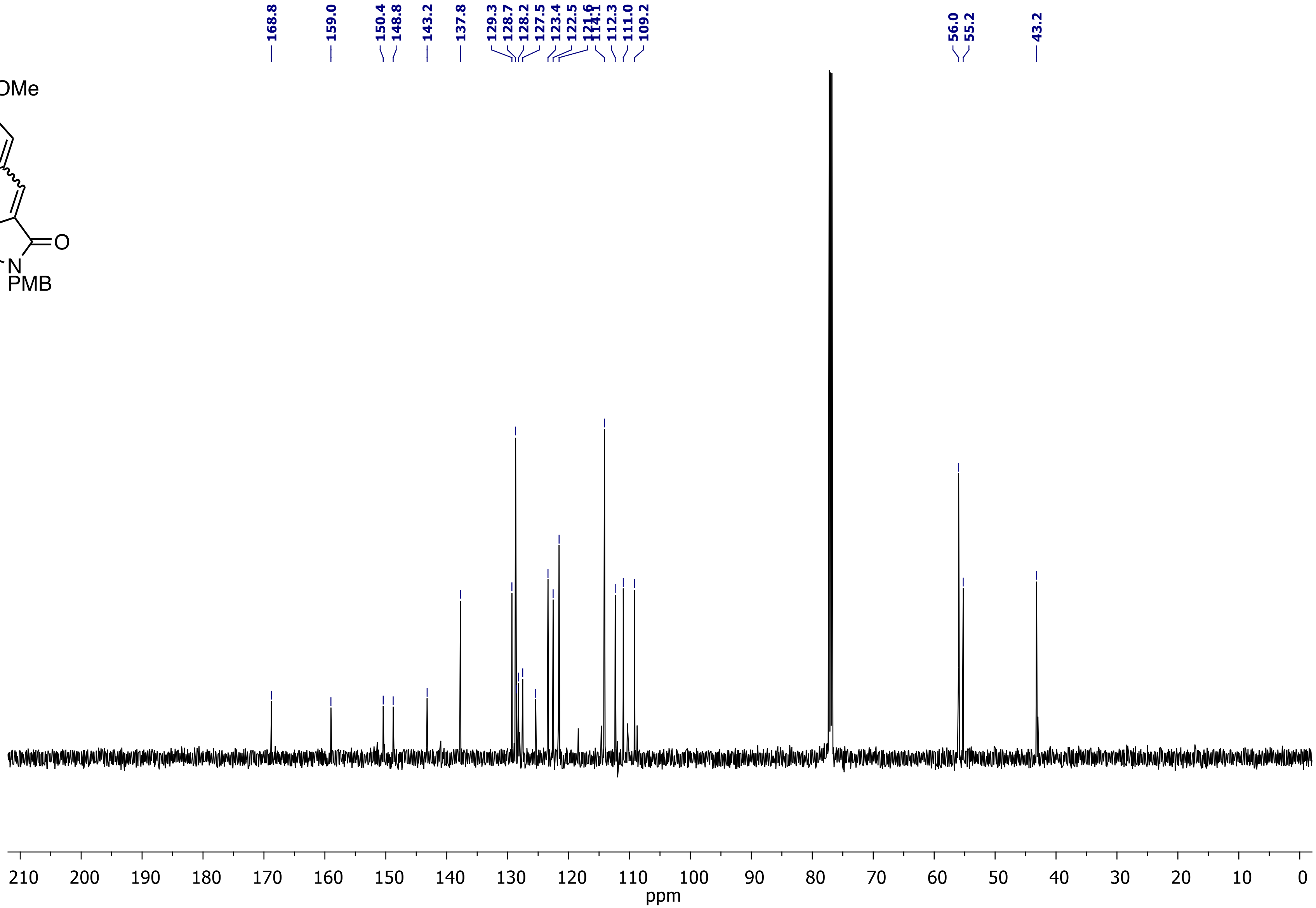

2-(2-Chlorophenyl)-1'-(4-methoxybenzyl)spiro[cyclopropane-1,3'-indolin]-2'-one (2a)

^{1}H NMR ($CDCl_3$, 600 MHz)

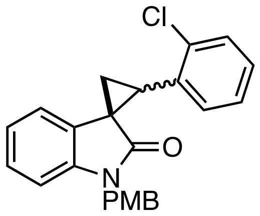

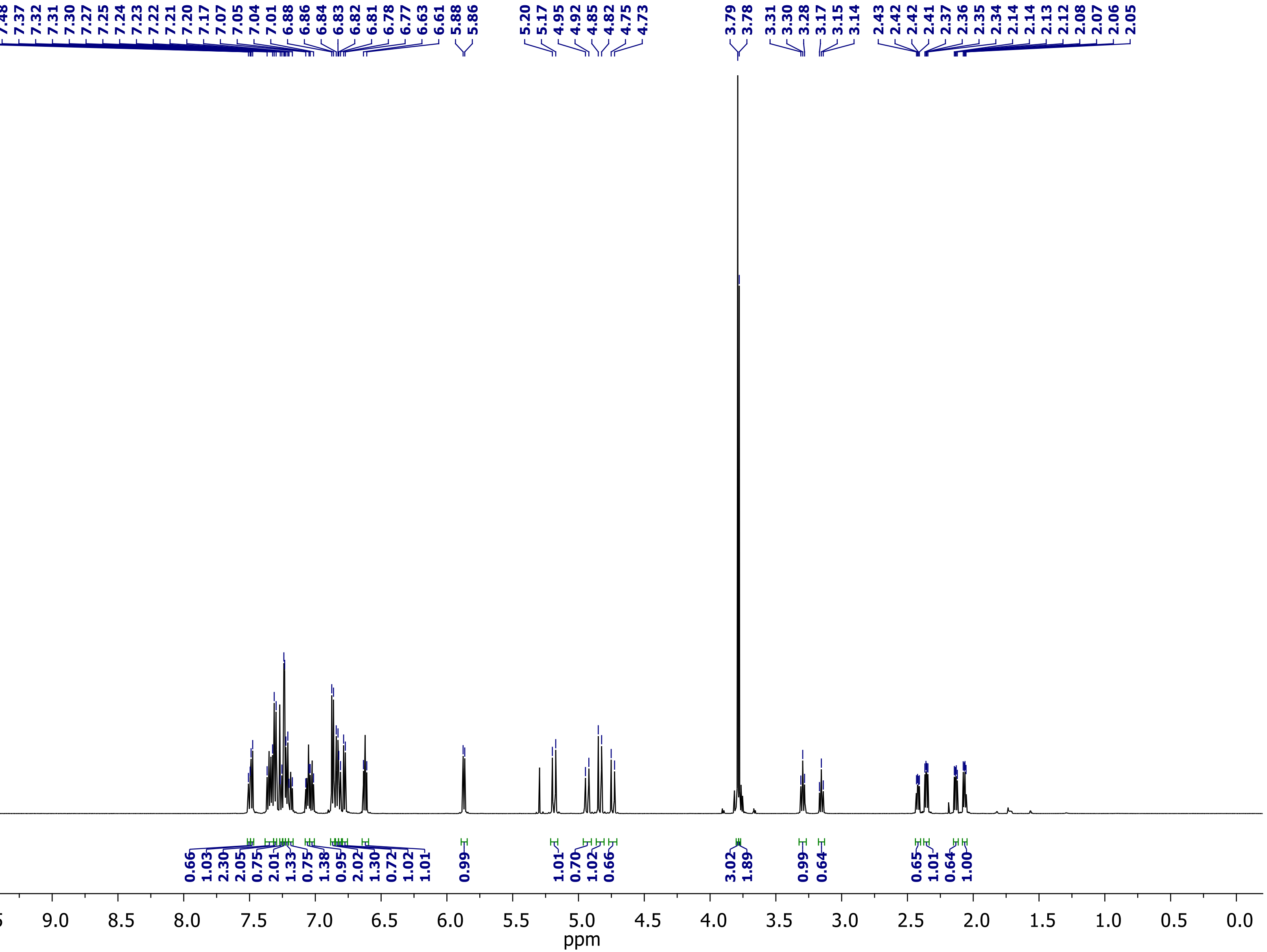

2-(2-Chlorophenyl)-1'-(4-methoxybenzyl)spiro[cyclopropane-1,3'-indolin]-2'-one (2a)

^{13}C NMR ($CDCl_3$, 150 MHz)

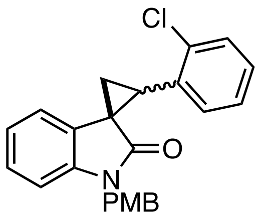

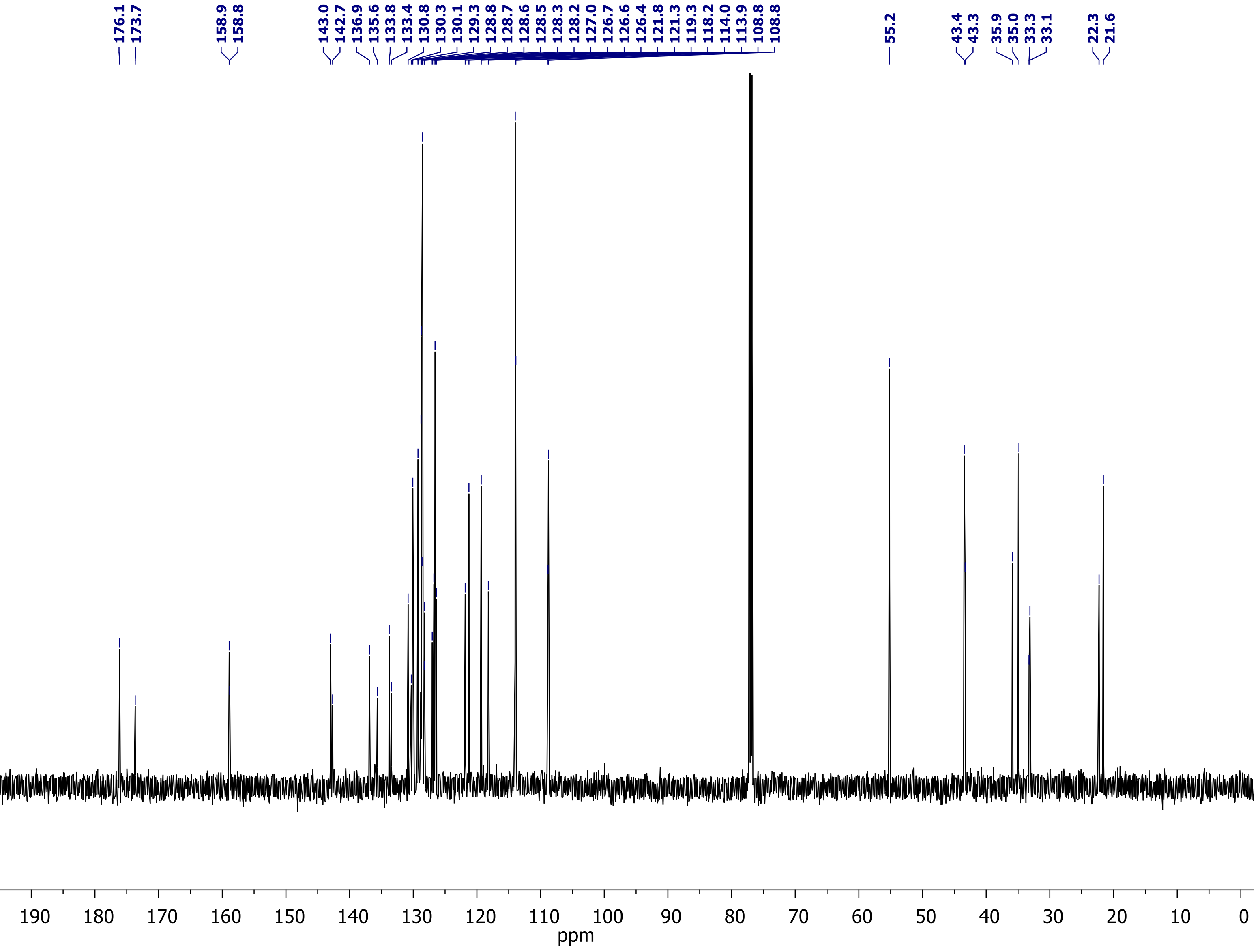

2-(3,4-Dimethoxyphenyl)-1'-(4-methoxybenzyl)spiro[cyclopropane-1,3'-indolin]-2'-one (2d)

^{1}H NMR ($CDCl_3$, 600 MHz)

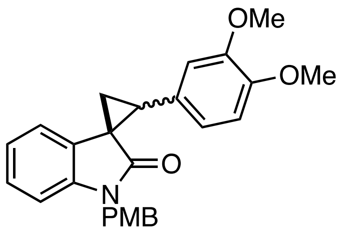

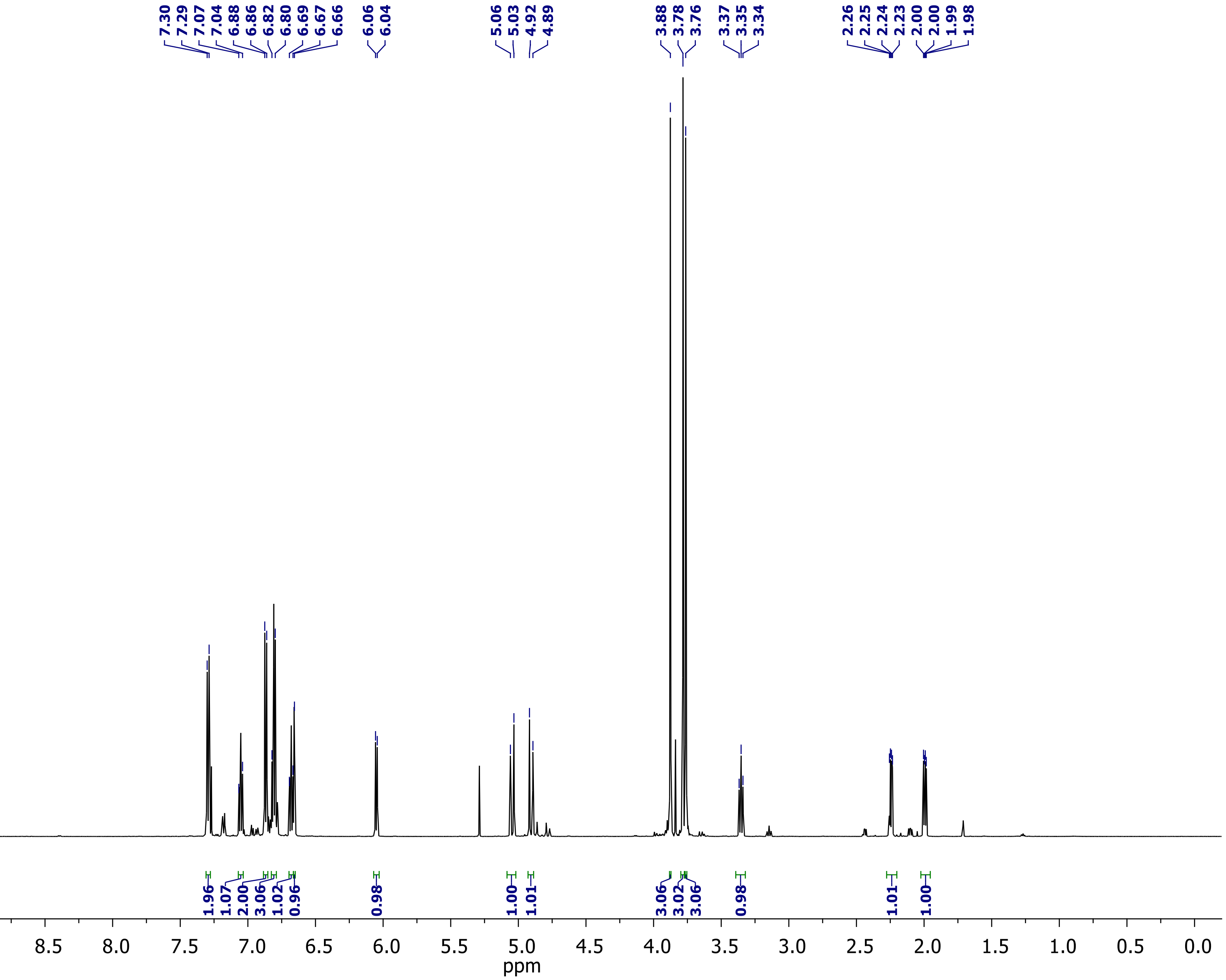

2-(3,4-Dimethoxyphenyl)-1'-(4-methoxybenzyl)spiro[cyclopropane-1,3'-indolin]-2'-one (2d)

^{13}C NMR ($CDCl_3$, 150 MHz)

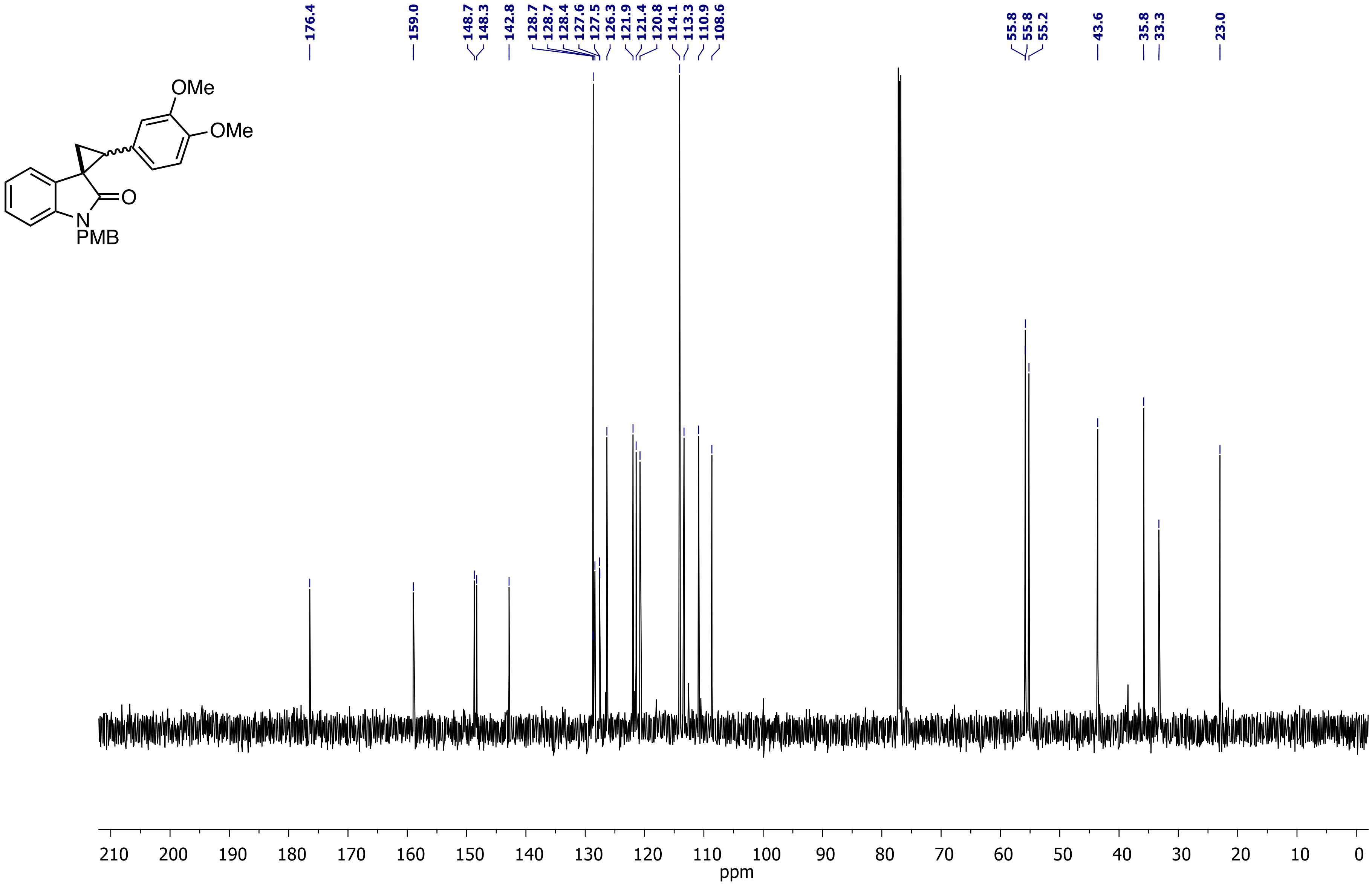

Supplement: Supplementary file 1 [file pharmaceutics-15-00228-s001.zip › pharmaceutics-2114157-supplementary.pdf]
